# Supplementary material for: Supervised and self-directed technology-based dual-task exercise training programme for older adults at risk of falling – Protocol for a feasibility study
Source: PLoS One. 2025 Mar 24;20(3):e0314829. doi: 10.1371/journal.pone.0314829 (PMC11932479; doi:10.1371/journal.pone.0314829)
Supplement: S2 Appendix — (DOCX) [file pone.0314829.s002.docx]

**Chief investigator: Dr Shin-Yi (Chloe) Chiou**

School of Sport, Exercise & Rehabilitation Sciences

The University of Birmingham

Edgbaston

Birmingham B15 2TT

Telephone: 0121 414 5315

Version 2, 09/04/2024; Study Identification Number for this trial:

**CONSENT FORM**

**Technology-based dual-task training in older adults**

**Please Initial Box**

1. I confirm that I have read and understand the Participant Information Sheet version __, date_________ for the above study and have had the opportunity to ask questions.
2. I confirm that I have had sufficient time to consider my participation in the study.
3. I understand that my participation is voluntary and that I am free to withdraw at any time without giving any reason, and without my medical care or legal rights being affected.
4. I understand that data collected up until the point that I withdraw may still be used.
5. I understand that sections of my medical notes may be looked at by responsible individuals from University of Birmingham, and/or the NHS Trust or from regulatory authorities where it is relevant to my taking part in research. I give permission for these individuals to have access to my records.
6. I agree to my data, including personal identifiable data, being stored on University of Birmingham computer systems.
7. I understand that my contact information will be held by the University of Birmingham and may be used to keep in touch with me for the purposes of the study.
8. I understand and acknowledge that data collected which identifies me by name e.g. informed consent forms, will be transferred from where it is collected to the study office at the University of Birmingham, where it will be held in strict confidence, password protected and encrypted for security. I give permission for the transfer and storage of this data.
9. I agree to the study interventions being monitored as described in the participant information sheet.
10. I agree my GP to be informed of my study participation.
11. I agree to take part in the focus group. I understand that my discussions in the focus groups will be audio recorded and transcribed by a member of the research team for the purpose of analysis and may be used as direct quotes in research publications, and that my identity will not be revealed.
12. I understand that all information collected from me for this study will be subject to the General Data Protection Regulation and Data Protection Act 2018. This information will be stored securely by the University of Birmingham, which is the data controller for the Study, for a minimum period of 10 years.
13. I agree to take part in this study.

1. ***Optional, please initial in either the ‘yes’ or ‘no’ box:** I agree to take part in the focus group. I understand that my discussions in the focus groups will be audio recorded and transcribed for the purpose of analysis and may be used as direct quotes in research publications, and that my identity will not be revealed.

Yes

No

1. ***Optional, please initial in either the `yes’ or `no’ box:** I agree to participant in the remote Time Up & Go (TUG) test.

No

Yes

________________ ________________ ____________________

Participant’s full name Date Participant’s signature

Email address:

_____________________ ________________ ____________________

Name of person taking consent Date Signature of person taking consent

*Original for the study file, a copy for the patient and a copy for the patient’s medical record*
