# Supplementary material for: Supervised and self-directed technology-based dual-task exercise training programme for older adults at risk of falling – Protocol for a feasibility study
Source: PLoS One. 2025 Mar 24;20(3):e0314829. doi: 10.1371/journal.pone.0314829 (PMC11932479; doi:10.1371/journal.pone.0314829)
Supplement: S3 Appendix — (PDF) [file pone.0314829.s003.pdf]

Technology-Based Dual-Task Training in Older Adults

EXERCISE HANDBOOK

Introduction

Instructions for home exercises:

- Please perform these exercises twice a week.

- Please ensure you are safe and comfortable to complete the exercises:
    - Have a drink of water close by,
    - Have support available for use throughout, eg. A chair or walking aid,
    - Wear loose, comfortable clothing,
    - Wear suitable, sturdy, flat footwear which fits securely to your feet.

- Have a timer available for each exercise (kitchen timer/stopwatch etc).

- Perform each exercise as stated – incorporating the appropriate level of progression for you as an individual.

- If you find an exercise easy, try to make it a little harder by trying one or more of the suggested alterations.

- If you find an exercise difficult, you can make it easier by trying one or more of the suggested alterations.

- Do not put yourself at risk – ensure you take rests and use support if needed.

- Stop doing the exercises if they cause pain, dizziness or breathlessness.

- Give your feedback to the Physiotherapist and Research Assistant at the group if you have any concerns about the exercises or call your support team.

CONTENTS

1. Static Exercises

1.1 Marching on the spot
1.2 Standing unsupported in tandem stand
1.3 Hip abduction
1.4 Hip extension
1.5 Squats
1.6 Tiptoe stand
1.7 Pendulum

2. Dynamic Exercises

2.1 Figure of eight walk
2.2 Walking forwards and backwards
2.3 Lunges
2.4 Functional reach
2.5 Toe tapping
2.6 Upper limb strength exercises
2.7 Side steps/ Simple grapevine

STATIC EXERCISES

(To be performed with a game from the PEAK app)

Prior to starting each exercise, ensure your device is set up on the music stand with the app open. Start a new game prior to beginning each exercise.

1.1 Marching on the spot

1.2 Standing unsupported in tandem stand

1.3 Hip abduction

1.4 Hip extension

1.5 Squats

1.6 Tiptoe stand

1.7 Pendulum

Marching on the spot

This exercise improves your strength and balance.

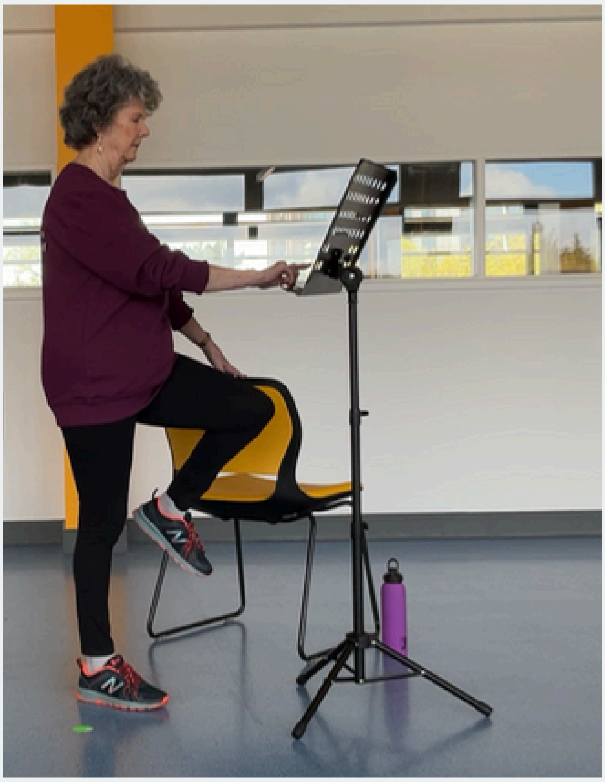

Stand with your feet shoulder-width apart with support available at one hand.

Start the game on the app and begin to play.

March on the spot at a relaxed pace trying to lift your knees to 90 degrees each time you march.

Continue this aiming for 2 minutes, have a 1-minute break and then do another 2 minutes as able.

If you find this easy:

1. Release hold of the support.

2. Increase the height you lift your knees.

3. Add ankle weights.

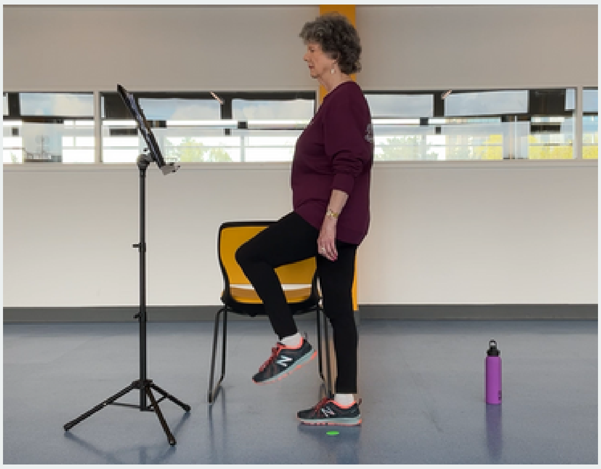

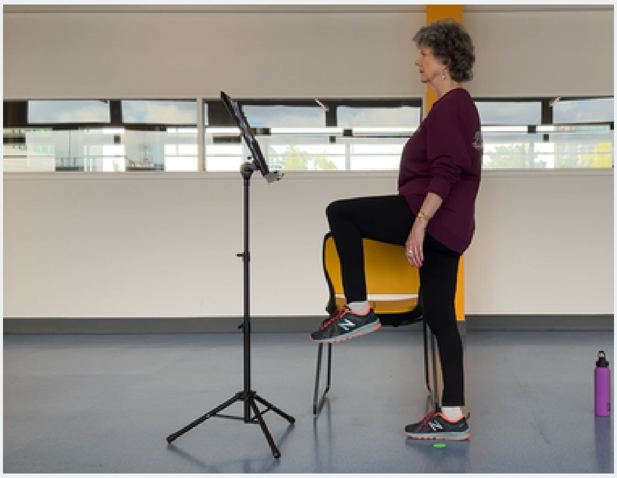

If you find this difficult:

1. Hold the support with 1 hand.

2. Decrease the height you lift your knees.

3. Do not use ankle weights.

Standing unsupported in tandem stand

This exercise improves balance.

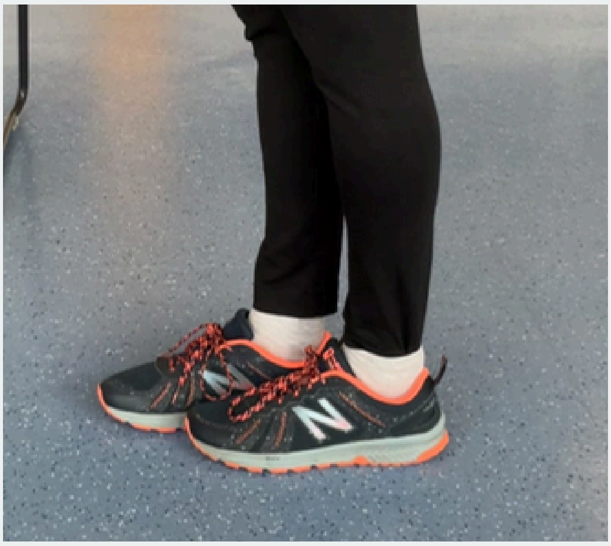

Semi-tandem stand

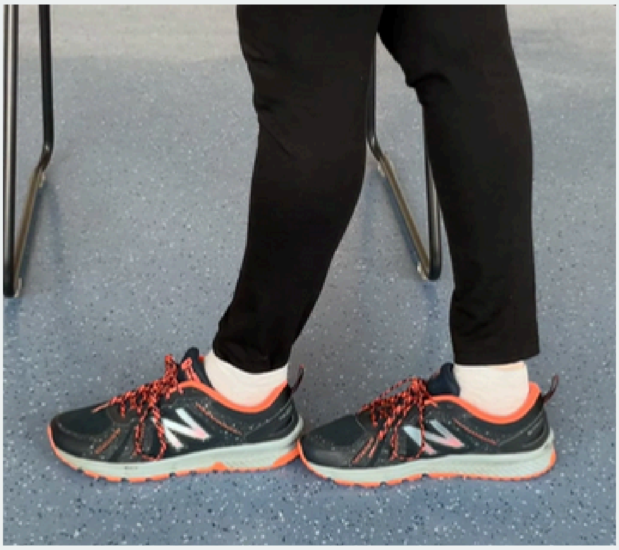

Full-tandem stand

Stand with your feet in a semi-tandem position – with feet together but one foot slightly in front of the other. Have support available at one hand.

Start the game on the app and begin to play.

Maintain your balance, releasing hold of the support if you can.

Stand in this way for as long as you can, alternating the front foot as needed.

Continue this aiming for 2 minutes, have a 1-minute break and then do another 2 minutes as able.

Hold onto the support available if required at any time during this exercise.

If you find this easy:

1. Release hold of the support.

2. Stand with your feet in full tandem (toe to heel) with or without support.

If you find this difficult:

1. Hold the support throughout.

2. Stand with your feet side by side.

Hip abduction

This exercise improves your strength and balance.

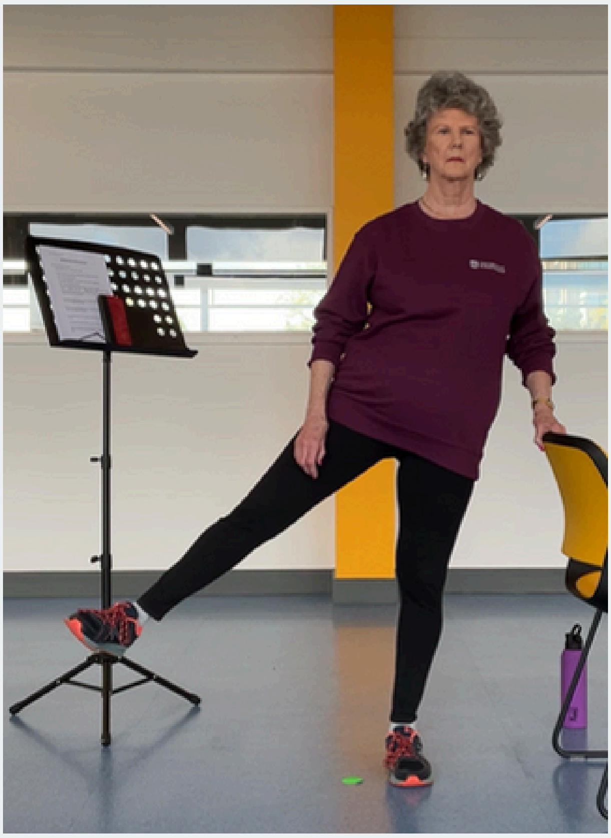

Stand with your feet shoulder-width apart, with support available at one hand.

Start the game on the app and begin to play.

Lift your leg slowly out to side keeping your knees and back straight, then return to starting position. Ensure you maintain your balance.

Repeat with each leg alternating. Have a break if required at any time.

Continue this aiming for 2 minutes, have a 1-minute break and then do another 2 minutes as able.

If you find this easy:

1. Release hold of the support.

2. Increase the height you lift your leg.

3. Add ankle weights.

If you find this difficult:

1. Hold the support throughout

2. Decrease the height you lift your leg

3. Do not use ankle weights

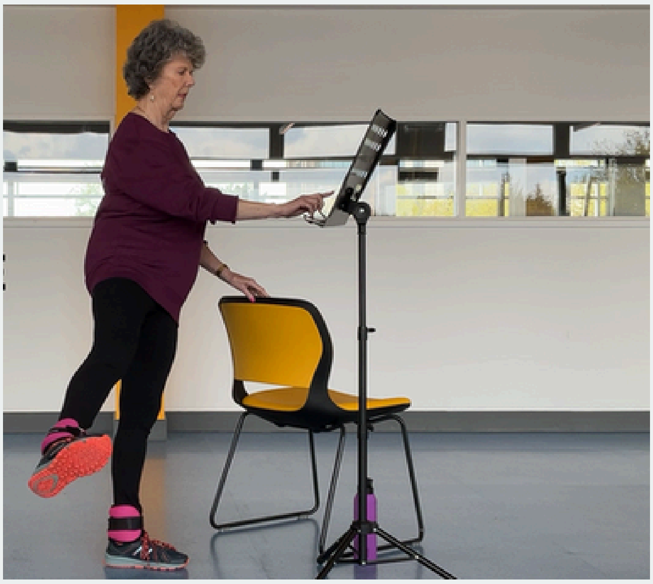

Hip extension

This exercise improves your strength and balance.

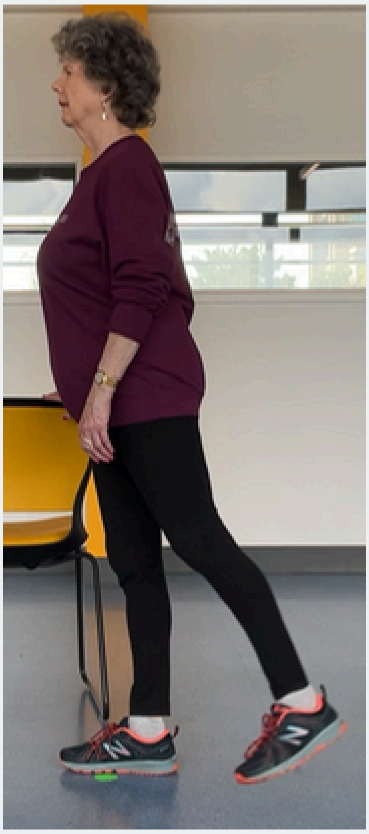

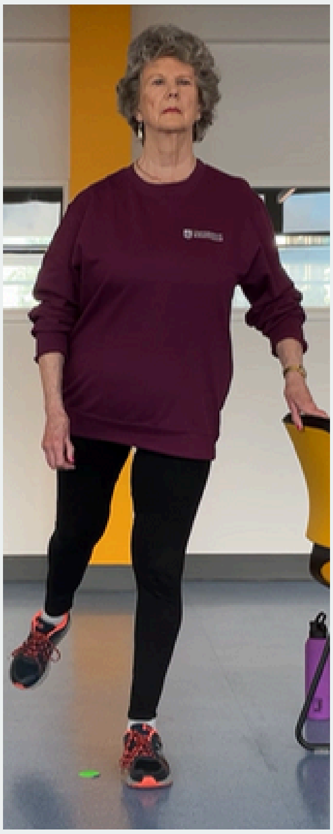

Stand with your feet shoulder-width apart, with support available at one hand.

Start the game on the app and begin to play.

Lift your leg out behind you, keeping your knees and back straight, then return to starting position. Ensure you maintain your balance.

Repeat with each leg alternating. Have a break if required at any time.

Continue this aiming for 2 minutes, have a 1 minute break and then do another 2 minutes as able.

If you find this easy:

1. Release hold of the support.

2. Increase the height you lift your leg.

3. Add ankle weights.

If you find this difficult:

1. Hold the support throughout.

2. Decrease the height you lift your leg.

3. Do not use ankle weights.

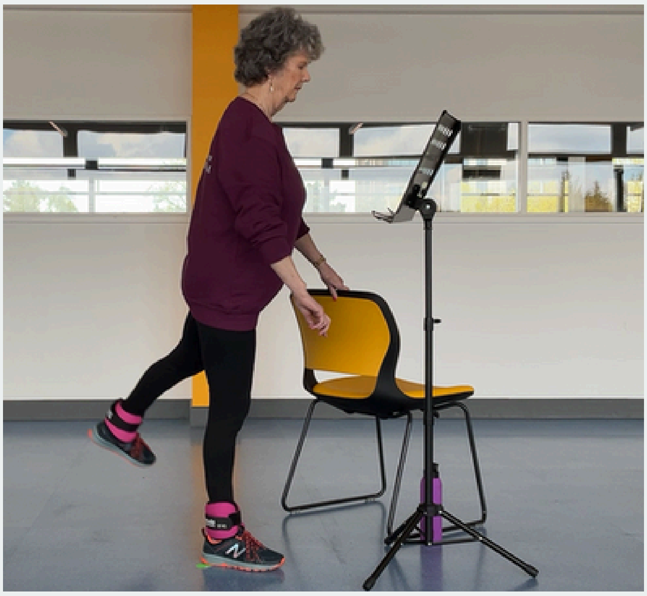

Squats

This exercise improves your strength.

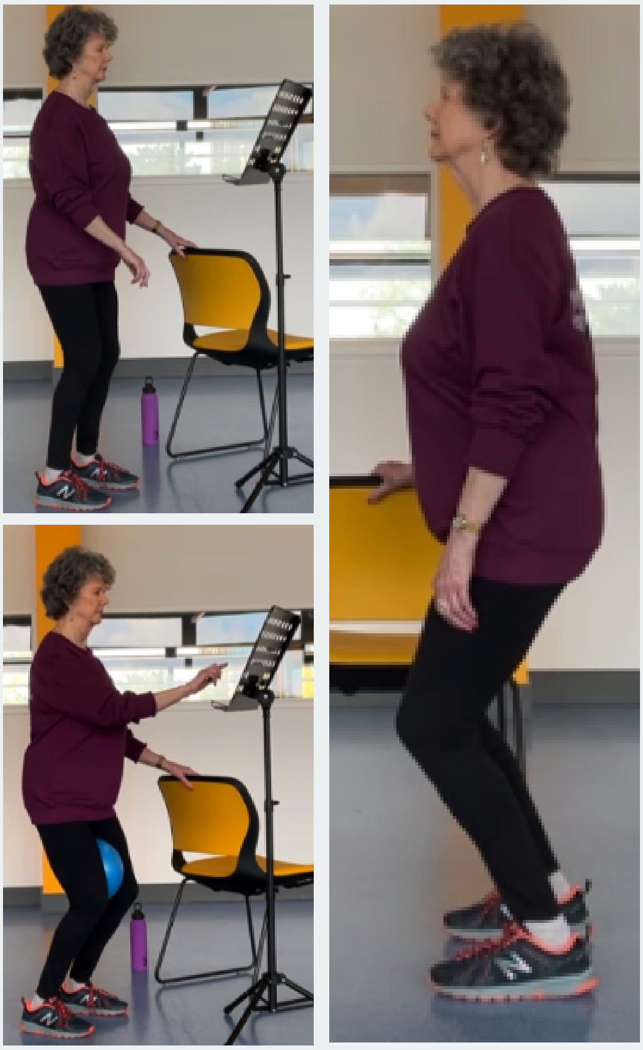

Stand with your feet shoulder-width apart, with support available at one hand.

Start the game on the app and begin to play.

Bend both knees slowly, aiming to achieve 45 degrees bend at the knee. Keep your back straight.

Hold for as long as you can then slowly return to starting position. Repeat. Have a break if required at any time.

Continue this aiming for 2 minutes, have a 1 minute break and then do another 2 minutes as able.

If you find this easy:

1. Release hold of the support.

2. Increase the bend at the knee.

3. Increase the time you hold the squat.

4. Add a ball between the knees.

If you find this difficult:

1. Hold the support throughout.

2. Decrease the bend at the knee.

3. Decrease the time you hold the squat.

4. Do not use ball between knees.

Tiptoe stand

This exercise improves your strength and balance.

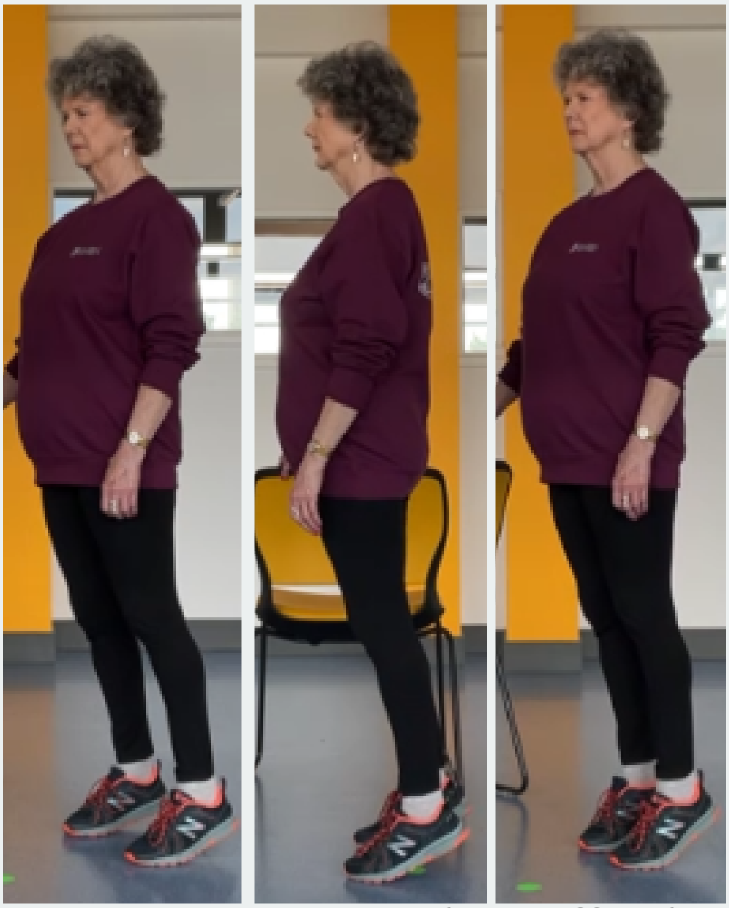

Easy Original Difficult

Stand with your feet shoulder-width apart, with support available at one hand.

Start the game on the app and begin to play.

Push up onto your tip toes on both feet at the same time.

Hold for as long as you can then return to starting position. Repeat.

Continue this aiming for 2 minutes, have a 1 minute break and then do another 2 minutes as able.

If you find this easy:

1. Release hold of the support.

2. Bring your feet closer together.

3. Increase the height you raise onto your toes.

4. Increase the time you stand on your toes.

If you find this difficult:

1. Hold the support throughout.

2. Stand with your feet further apart.

3. Decrease the height you raise onto your toes.

4. Decrease the time you stand on your toes.

Pendulum

This exercise improves your balance.

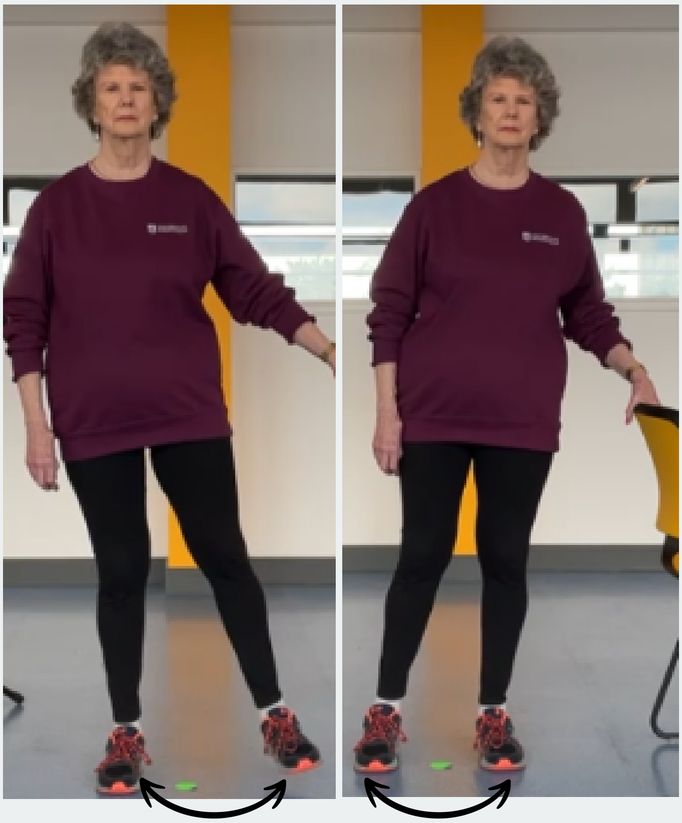

Stand with your feet shoulder-width apart, with support available at one hand.

Start the game on the app and begin to play.

Gently sway side to side slowly taking your body weight from one leg to the other.

If able – lift 1 leg at a time until you are standing on the other leg for short periods at a time.

Hold for as long as you can. Repeat with each leg alternating

Continue this aiming for 2 minutes, have a 1 minute break and then do another 2 minutes as able.

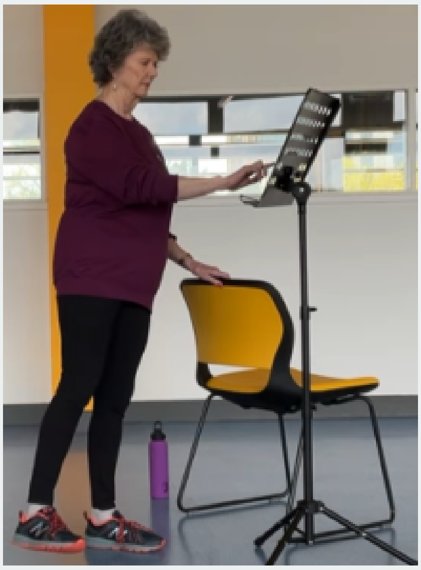

If you find this easy:

1. Release hold of the support

2. Increase the length of time stood on 1 leg.

If you find this difficult:

1. Hold onto the support throughout.

2. Decrease the length of time stood on 1 leg.

3. Sway side to side only (no single leg stand).

DYNAMIC EXERCISES

(To be performed without the app)

2.1 Figure of eight walk

2.2 Walking forwards and backwards

2.3 Lunges

2.4 Functional reach

2.5 Toe-tapping

2.6 Upper limb strength exercises

2.7 Side-steps/simple grapevine

Figure of eight walk

This exercise improves your postural stability and lower limb strength.

Stand with your current walking aid in a clear space in your home.

Walk in figure of 8 at a relaxed pace using the aid if needed.

Repeat for 2 minutes.

If you find this easy:

1. Stop using your aid if able.
2. Increase your walking speed.
3. Add ankle weights.

If you find this difficult:

1. Use a walking aid.
2. Decrease your walking speed.
3. Do not use ankle weights.

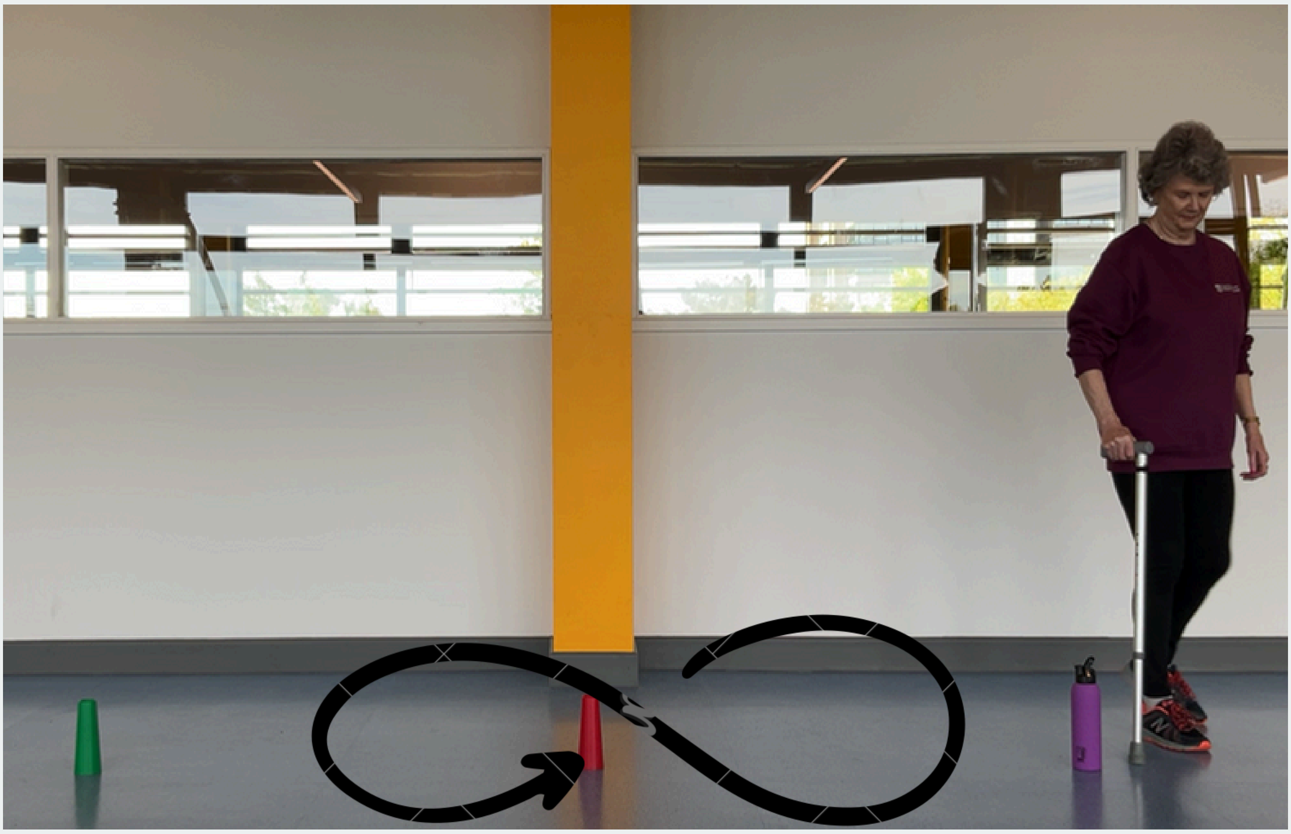

Walking forwards and backwards

This exercise improves your postural stability and lower limb strength.

Find a clear and straight area in your home with your current walking aid.

Walk forward 5 steps at a relaxed pace using your current walking aid.

Now walk backwards 5 steps carefully and at a relaxed pace using your current walking aid.

Repeat for 2 minutes forwards and backwards.

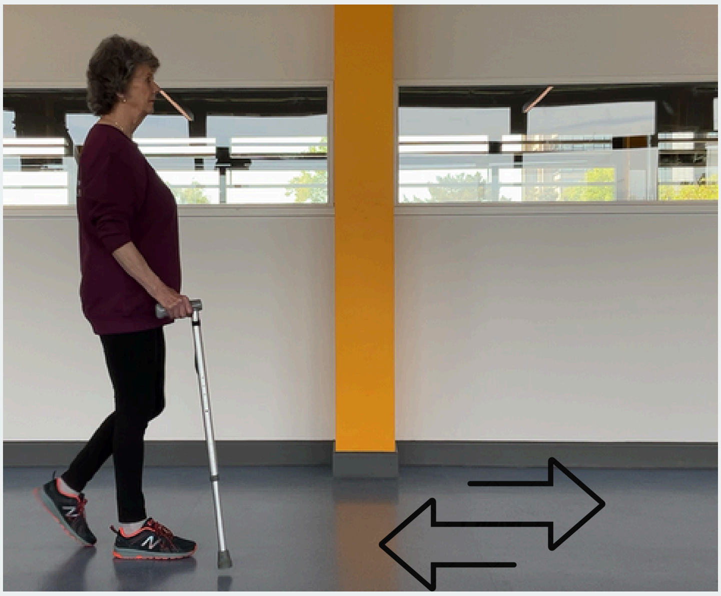

If you find this difficult:

1. Use a walking aid.

2. Decrease walking speed.

3. Do not use ankle weights.

4. Do not include backward walking, instead turn and walk forward in the other direction.

If you find this easy:

1. Try without using your aid.

2. Increase walking speed.

3. Add ankle weights.

4. Try tightrope walking with one foot directly in front of the other.

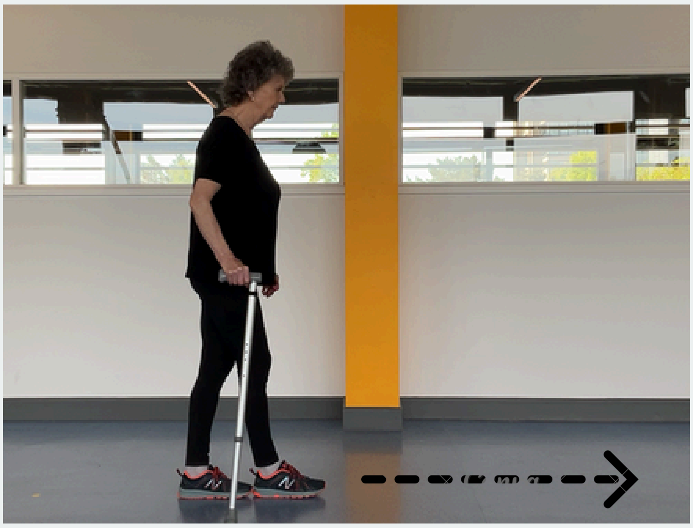

Lunges

This exercise improves your lower limb strength.

Stand in a comfortable position with your feet shoulder-width apart and support available at one side of you.

Take a step forward and put your weight through the front leg with your front knee bent. You will find your back heel raises off the floor.

Hold for 10 seconds before you return to starting position.

Repeat with the other leg.

Repeat for 2 minutes with alternating legs in front.

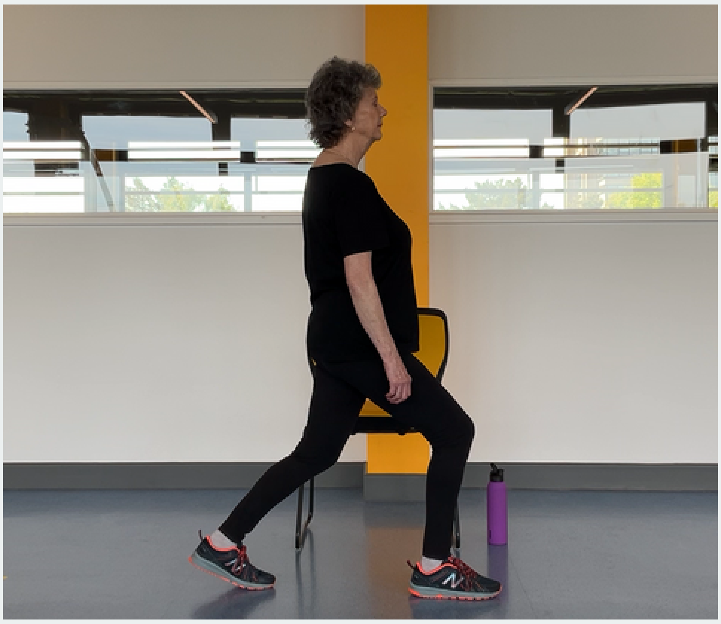

If you find this difficult:

1. Use support.

2. Decrease distance of lunge.

3. No ankle weights.

If you find this easy:

1. Do not use a support.

2. Increase distance of lunge.

3. Add ankle weights.

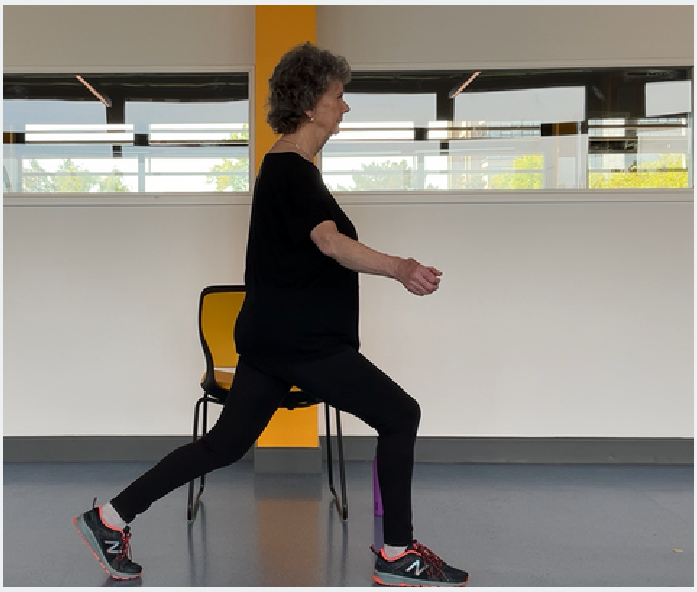

Functional Reach

This exercise improves your postural stability and lower limb strength.

Find a space in your home next to a clear wall or by the kitchen counter/cupboards.

Stand in a comfortable position with your feet shoulder-width apart and support available.

With one arm at a time, reach forward to touch an imaginary target on the wall/cupboard door. Repeat reaching in other directions – above your head, out to each side, across your body.

If able, turn 90 degrees and reach round behind you to touch imaginary targets at different heights.

Now step back and repeat to extend your reach. You should find that you lean through 1 leg more than the other, or that you stand on slight tip toes to really extend your reach.

Repeat for 2 minutes.

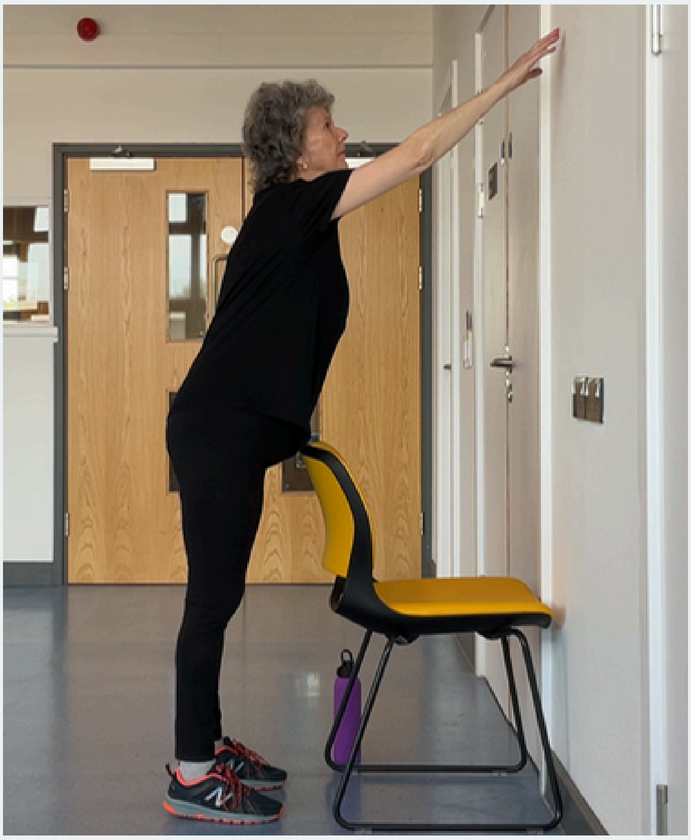

If you find this easy:

1. Do not use any support.

2. Increase distance of target to reach further.

3. Move your feet closer together.

If you find this difficult:

1. Use a support or sit down.

2. Decrease distance of target.

3. Move your feet further apart.

Toe Tapping

This exercise improves your postural stability and lower limb strength.

Stand in a comfortable position with your feet shoulder-width apart and support available.

Lean to one side so that your body weight is through that leg - use the support if needed.

Lift the other foot and tap your toes forwards 3 times. Now return to starting position and regain your balance.

Repeat by tapping the same foot 3 times out to the side and 3 times behind you.

Repeat with the other leg.

Repeat for 2 minutes with alternating legs.

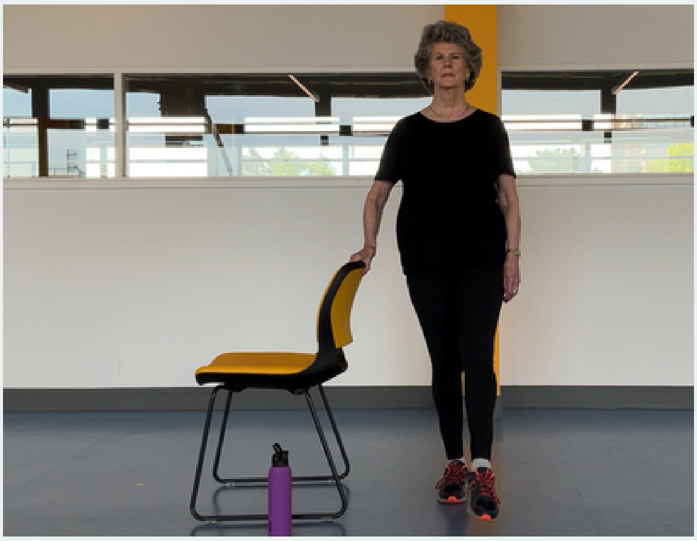

If you find this difficult:

1. Perform holding a support.

2. Do not lift your foot off the floor fully to move – slide across floor in each direction.

If you find this easy:

1. Perform without holding a support.

2. Repeat without bringing your foot back to centre in between.

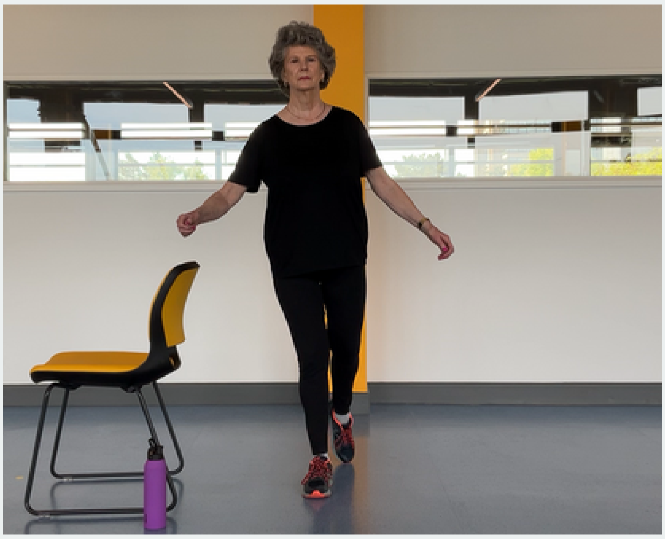

Upper Limb Strength Exercises

This exercise improves your postural stability and lower limb strength.

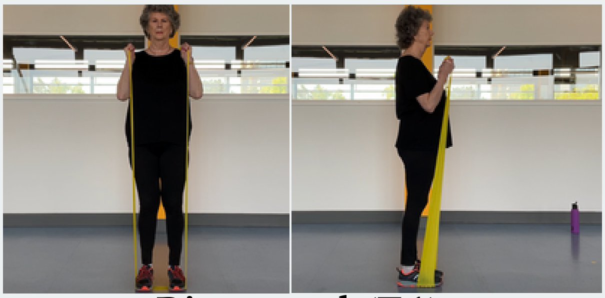

Bicep curl (**E1**)

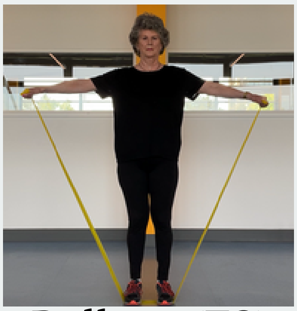

Pull-up (**E2**)

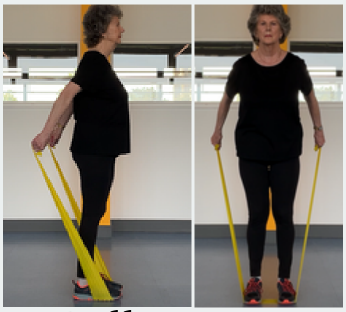

Pull-up (**E3**)

If you find this easy:

1. If sitting, progress to standing.

2. Increase the number of repetitions.

3. Increase the resistance of band.

4. Increase the distance of pull .

If you find this difficult:

1. If standing, return to sitting.

2. Decrease the number of repetitions.

3. Decrease the resistance of band.

4. Decrease the distance of pull.

Stand in a comfortable position with your feet shoulder-width apart and support available.

Place the band flat on the floor infront of you and step onto it with both feet.

Hold each end of the band in each hand. Ensure that you take up the slack by wrapping the band around each hand as needed.

NB If unable to stand without support – sit down on a chair and place the band under both feet, holding each end of the band in each hand.

E1. Tuck your elbows in at 90 degrees and pull up towards you bending at the elbows (bicep curls) x10

E2. Take both arms out to the side with straight elbows and pull up x 10

E3. Stand with knees slightly bent and arms loosely by your side. Pull up by bending your elbows as high as you can x10

Continue these exercises in rotation for 2 minutes.

Side-Steps/ Simple Grapevine

This exercise improves your postural stability.

Find a clear and straight area in your home with your current walking aid.

Walk sideways for 5 steps at a relaxed pace. Walk sideways back the other way for 5 steps at a relaxed pace.

If able, progress to a simple grapevine by crossing one leg forwards over the other and stepping the back leg next to it for 5 steps, repeat back the other way for 5 steps.

Repeat for 2 minutes.

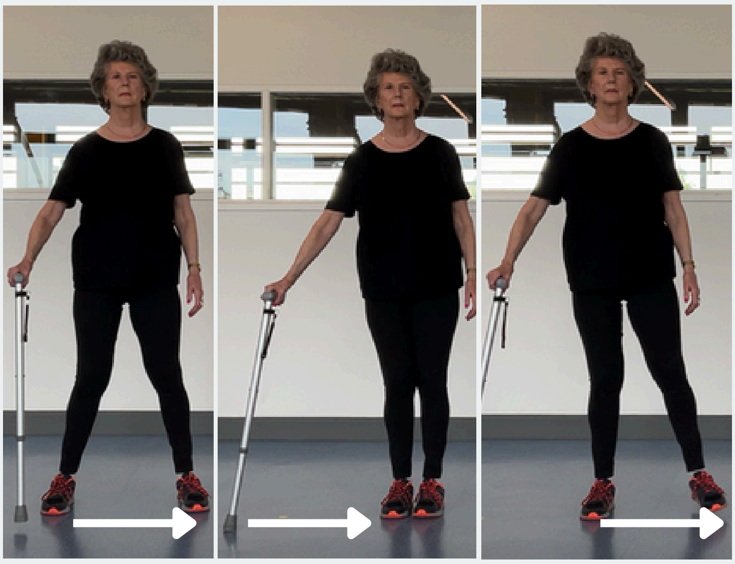

If you find this difficult:

1. Hold a support throughout.

2. Decrease the speed of sideways stepping.

3. Decrease the step size.

4. Continue with sideways walk, do not progress to grapevine (no crossing of feet).

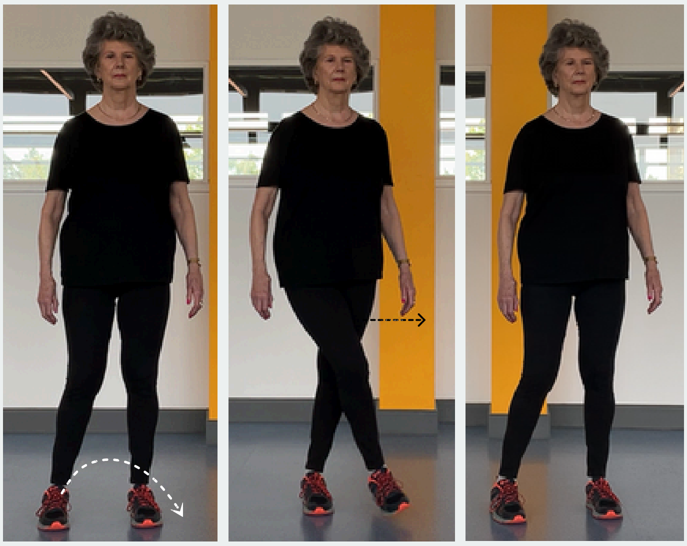

If you find this easy:

1. Do not hold a support.

2. Increase the speed of sideways stepping.

3. Increase step size.

4. Progress to grapevine (crossing feet.

Contact Us

If you would like further information or any assistance, please contact:

Prerna Mathur
Tel. no. - 07436231211
Email - p.s.mathur@bham.ac.uk
