# Supplementary material for: Supervised and self-directed technology-based dual-task exercise training programme for older adults at risk of falling – Protocol for a feasibility study
Source: PLoS One. 2025 Mar 24;20(3):e0314829. doi: 10.1371/journal.pone.0314829 (PMC11932479; doi:10.1371/journal.pone.0314829)
Supplement: S4 Appendix — (PDF) [file pone.0314829.s004.pdf]

PEAK-BRAIN TRAINING APP

HANDBOOK

Technology-Based Dual-Task Training in Older Adults

Introduction

Training the brain and the body together has been shown to improve balance and reduce the risk of falling. This intervention combines cognitive and physical exercises to enhance the ability to multitask. Our research project will examine how acceptable this new programme is likely to be to older people at risk of falling and will evaluate the feasibility of delivering it within the NHS.

The games on the PEAK app are helpful in training the mind. It offers a set of brain-training games and a unique method of playing them which are both scientifically designed and fun to play. It is important to do a range of cognitive training and train multiple skills such as focus, memory, mental agility and problem-solving. You will be advised on which games to play each week and these will be a combination to ensure multiple skills are put to the test.

This mind-body training program is of 24 weeks. There will be in-person exercise classes weekly for the first 12 weeks of the program to get you acquainted with the physical exercises and the games on the PEAK app. You will be taught three new games and exercises weekly in these classes which you can repeat at home two times per week. This handbook will introduce you to 18 games which have been deemed suitable to use during the study, but initially, you will perform 3 games per week and you will be introduced to them gradually in class to ensure you are comfortable with them.

After the 12 weeks of group input, you will be asked to continue at home for another 12 weeks incorporating everything you have learnt in class and performing both physical and cognitive exercises.

Introduction

18 games have been selected from the PEAK app for this program. These selected games train the following skills:

1. Focus
Focus contains all the abilities that are based on our attention capacity. Sustained attention is the ability to maintain concentration over long periods of time.

2. Memory
Memory is a mental process by which you store information that you can then recall at a later time.

3. Mental Agility
Your mental agility is defined by how well you are able to manage changes, adapt to new situations, and handle multi-tasks at the same time.

4. Problem solving
This mental process is involved in finding solutions to a problem. We use this skill in our everyday life almost everywhere.

CONTENTS

1. Focus

1.1 Must Sort
1.2 Rush Back
1.3 Unique
1.4 Decoder

2. Memory

2.1 Perilous Path
2.2 Memory Sweep
2.3 Spin Cycle
2.4 Apprentice Wizard
2.5 Partial Match

3. Mental Agility

3.1 True Color
3.2 Face Switch
3.3 Refocus
3.4 Turtle Traffic

4. Problem- Solving

4.1 Puzzle Box
4.2 Slider
4.3 Low Pop
4.4 Castle Block
4.5 Earth Defense

FOCUS

1.1 Must Sort

1.2 Rush Back

1.3 Unique

1.4 Decoder

Must Sort - Focus

Sort the items correctly by tapping on the left or right side of the screen.

Wrongly sorted items give you a time penalty.

Rush Back - Focus

Memorize the card.

Does the current card match the card that came before it?

Unique - Focus

Observe the different shapes on the screen.

Find the odd one out, and tap on it.

Decoder - Focus

You must memorize all of the three-digit codes on the phone.

The goal is to spot when the phone code appears on the building.

You must focus and wait for the digits to appear in the exact order.

When you spot the last digit of the code sequence, tap anywhere on the screen.

Tap before the next digit appears, the faster you tap the bigger your bonus.

MEMORY

2.1 Perilous Path

2.2 Memory Sweep

2.3 Spin Cycle

2.4 Apprentice Wizard

2.5 Partial Match

Perilous Path - Memory

Memorise the position of the danger tiles...

... and avoid them when connecting the dots!

Memory Sweep - Memory

Memorise the highlighted tiles...

...and remember their positions when they are gone.

Spin Cycle - Memory

Observe the changing patterns.

Is the statement true or false compared to the round before.

Apprentice Wizard - Memory

Memorise where each symbol appears.

Then drag the symbols to their original location.

Partial Match - Memory

Observe the shape.

Does the current shape match the shape that came before it?

Tap on “PARTLY” if one or two of the following have changed: shape, color, rotation.

MENTAL AGILITY

3.1 True Color

3.2 Face Switch

3.3 Refocus

3.4 Turtle Traffic

True Color - Mental Agility

A word and a color will appear on the cards.

Determine if the word at the top matches the color at the bottom.

Try to ignore the meaning of the word at the bottom and focus on just its color.

Face Switch

Mental Agility

Determine if the woman on the top card is happy...

...or if the man on the bottom card is wearing glasses.

Refocus - Mental Agility

Determine if the number on the top card is even...

...or if the letter on the bottom card is vowel.

Turtle Traffic - Mental Agility

Tap anywhere on the screen to move the turtle up and release to let it drop.

Eat jellyfish for points, but avoid plastic bags, fishing nets and spikey things!

Look out for orbs - they score big points.

PROBLEM SOLVING

4.1 Puzzle Box

4.2 Slider

4.3 Low Pop

4.4 Castle Block

4.5 Earth Defense

Puzzle Blox - Problem Solving

Match the target at the top by tapping away blocks you don’t need.

Blocks will fall down if not supported.

Slider - Problem Solving

Slide blocks to create a path between the end points.

Fewer moves and high speed earns high score.

Low Pop- Problem Solving

Tap the tiles in order of lowest to the highest.

Castle Block - Problem Solving

Fill the grid at the top using all the pieces.

Touch, drag and release pieces to place them or reposition.

Tap pieces to remove them quickly.

Earth Defense - Problem Solving

Tap to place mines and protect Earth from incoming meteors.

Score big by taking out multiple meteors with one mine.

The bigger the mine when hit, the bigger the score.

Be precise! You can place 2 mines at once and they vanish after 2 seconds.
