# Supplementary material for: Supervised and self-directed technology-based dual-task exercise training programme for older adults at risk of falling – Protocol for a feasibility study
Source: PLoS One. 2025 Mar 24;20(3):e0314829. doi: 10.1371/journal.pone.0314829 (PMC11932479; doi:10.1371/journal.pone.0314829)
Supplement: S5 Appendix — (DOCX) [file pone.0314829.s005.docx]

**Appendix 5. Participants’ Exit questionnaire to be filled at 12 and 24 weeks**

| **Questions** | **Possible answers** |
| --- | --- |
| Overall, how beneficial did you find the training to be? | 1 (not at all) to 10 (very much so) |
| How easy was it to fit the training into your daily routine? | 1 (not at all) to 10 (very much so) |
| Which of the following did you enjoy about the app? | - Fun to do - Easy to setup - Ability to do it at home, any time I want - Sense of achievement after doing it - Energise me and uplifting my spirit - Other........ |
| Which of the following limited your use of the app? Tick all that apply | - Difficult to set up the device for exercise - Exercise is too easy for me - Can’t find a space to do it - Didn't enjoy the activities/instructors/app - Lack of variety in exercises - Just forgot - Didn't feel enough progression/benefits through the exercise - Didn’t have time to do all the exercises in a week - Exercises too physically challenging - Other........ |
| How happy were you with the level of improvement of balance between the start and finish? | 1 (disappointed) to 10 (very happy) |
| Have you noticed any other physical benefits or improvement after completing 6 months of the blended DT training? | Yes/No/Not sure  If yes, please specify |
| Were you happy with the way your data being stored and collected by the PEAK? | Yes/No/Not sure  If no, please specify |
| Do you think the blended DT training programme with PEAK is a good way of achieving the above? | Yes/No/Not sure  If no or not sure, please specify |
| Did you receive the support you needed during the programme? | Yes/No/Not sure  If no or not sure, please specify |
| How likely will you continue with the training after the study completion? | 1 (not at all) to 10 (very much so) |
| If you were to continue with the training, how regularly do you think you would do it? | • Every day  • A couple of times a week  • Just occasionally  • Whenever I felt like my balance needed a boost  • I don't think I would use it again |
| What could we have changed or improved? | - |
