# Supplementary material for: Supervised and self-directed technology-based dual-task exercise training programme for older adults at risk of falling – Protocol for a feasibility study
Source: PLoS One. 2025 Mar 24;20(3):e0314829. doi: 10.1371/journal.pone.0314829 (PMC11932479; doi:10.1371/journal.pone.0314829)
Supplement: S1 File — (DOCX) [file pone.0314829.s006.docx]

## Full/long title of the study

Feasibility and acceptability of a supervised and self-directed technology-based Dual Task training programme for older adults at risk of falling.

## Short study title/acronym

Technology-based dual-task training in older adults

## Protocol version number and date

Version 03. 21/05/2024

## Research reference numbers

| **IRAS Number** | 327056 |
| --- | --- |
| **Sponsor reference number** | ERN_22-1294 |
| **ISRCTN number** | 15123197 |
| **REC reference number** | 24/EE/0059 |

This protocol has regard for the HRA guidance

## Signature page

The undersigned confirm that the following protocol has been agreed and accepted and that the Chief Investigator agrees to adhere to the signed University of Birmingham’s Sponsorship CI declaration.

I agree to ensure that the confidential information contained in this document will not be used for any other purpose other than the evaluation or conduct of the investigation without the prior written consent of the Sponsor

I also confirm that I will make the findings of the study publically available through publication or other dissemination tools without any unnecessary delay and that an honest accurate and transparent account of the study will be given; and that any discrepancies from the study as planned in this protocol will be explained.

Chief Investigator:

Signature: ................................................................................. Date: ......../……...../…….....

Name: (please print): Shin-Yi Chiou

## Sponsor statement:

Where the University of Birmingham takes on the sponsor role for protocol development oversight, the signing of the IRAS form by the sponsor will serve as confirmation of approval of this protocol.

##

## Table of Contents

[Full/long title of the study 1](#_Toc158892604)

[Short study title/acronym 1](#_Toc158892605)

[Protocol version number and date 1](#_Toc158892606)

[Research reference numbers 1](#_Toc158892607)

[Signature page 1](#_Toc158892608)

[Sponsor statement: 1](#_Toc158892609)

[Table of Contents 2](#_Toc158892610)

[Key study contacts 3](#_Toc158892611)

[Study summary 4](#_Toc158892612)

[Funding and support in kind 4](#_Toc158892613)

[Role of study sponsor and funder 4](#_Toc158892614)

[Roles and responsibilities of study management committees/groups and individuals 4](#_Toc158892615)

[Protocol contributors 4](#_Toc158892616)

[Study flow chart 5](#_Toc158892617)

[Study protocol 5](#_Toc158892618)

[1. Background 5](#_Toc158892619)

[2. Rationale 6](#_Toc158892620)

[3. Theoretical framework 6](#_Toc158892621)

[4. Research question/aims 6](#_Toc158892622)

[4.1. Research aim 6](#_Toc158892623)

[4.2. Research questions 6](#_Toc158892624)

[4.3. Outcomes 7](#_Toc158892625)

[5. Study design and methods of data collection and data analysis 7](#_Toc158892626)

[5.1. Study design 7](#_Toc158892627)

[5.2. Recruitment 7](#_Toc158892628)

[5.3. Planned interventions 8](#_Toc158892629)

[5.4. Assessments 11](#_Toc158892630)

[5.5. Statistical analysis 13](#_Toc158892631)

[6. Study setting 13](#_Toc158892632)

[7. Participant recruitment 13](#_Toc158892633)

[7.1. Eligibility Criteria 14](#_Toc158892634)

[7.1.1. Inclusion criteria 14](#_Toc158892635)

[7.1.2. Exclusion criteria 14](#_Toc158892636)

[7.2. Recruitment target 14](#_Toc158892637)

[7.2.1. Size of recruitment target 14](#_Toc158892638)

[7.2.2. Recruitment technique 14](#_Toc158892639)

[7.3. Recruitment 15](#_Toc158892640)

[7.3.1. Participant identification 15](#_Toc158892641)

[7.3.2. Consent 15](#_Toc158892642)

[8. Safety reporting 15](#_Toc158892643)

[9. Data Handling and Record Keeping 16](#_Toc158892644)

[Source data 16](#_Toc158892645)

[Data collection 17](#_Toc158892646)

[Participant completed questionnaires 17](#_Toc158892647)

[Data security 17](#_Toc158892648)

[Archiving 18](#_Toc158892649)

[10. Ethical and regulatory considerations 18](#_Toc158892650)

[10.1. Assessment and management of risk 18](#_Toc158892651)

[10.2. Research Ethics Committee (REC) and other Regulatory review & reports 19](#_Toc158892652)

[Regulatory Review & Compliance 19](#_Toc158892653)

[Amendments 19](#_Toc158892654)

[10.3. Peer review 19](#_Toc158892655)

[10.4. Patient & Public Involvement 19](#_Toc158892656)

[10.5. Protocol compliance 19](#_Toc158892657)

[10.6. Data protection and patient confidentiality 19](#_Toc158892658)

[10.7. Indemnity 20](#_Toc158892659)

[10.8. End of study and archiving 20](#_Toc158892660)

[10.9. Access to the final study dataset 20](#_Toc158892661)

[10.10. Dissemination policy 20](#_Toc158892662)

[10.11. Authorship eligibility guidelines and any intended use of professional writers 21](#_Toc158892663)

[11. References 21](#_Toc158892664)

[12. Appendices 22](#_Toc158892665)

[12.1. Appendix 1- Required documentation 22](#_Toc158892666)

[12.2. Appendix 2 – Schedule of Procedures 22](#_Toc158892667)

[12.3. Appendix 3 – Amendment History 22](#_Toc158892668)

## Key study contacts

Insert full details (including phone and email numbers, as applicable) of the key study contacts, including the following:

Chief Investigator: Dr Shin-Yi Chiou

Study Co-ordinator: N/A

Sponsor: University of Birmingham

Joint-sponsor(s)/co-sponsor(s):N/A

Funder(s): The National Institute for Health and Care Research (NIHR)

Key Protocol Contributors: Dr Shin-Yi Chiou, Dr Magdalena Chechlacz, Professor Afroditi Stathi, Dr Victoria Goodyear, Dr Laura Magill, Mrs Natalie Rowland (nee Ives), Ms Angela Cooper, Dr Daisy Wilson, Dr Caroline Miller, Helen Thomas, Emily Clements, and Dr Philip Kinghorn.

## Study summary

Study Title: Feasibility and acceptability of a supervised and self-directed technology-based Dual Task training programme for older adults at risk of falling.

Internal ref. no. (or short title): Dual-task training in older adults.

Study Design: A single-arm, non-randomised feasibility study.

Study Participants: adults aged 65 years and above.

Planned Size of recruitment target (if applicable): 50 adults aged 65 years and above.

Follow up duration (if applicable) No follow-up in this study.

Planned Study Period: 24 months.

Research Question/Aim(s): This study aims to examine how feasible it is to deliver a blended supervised and self-directed technology-based dual-task training programme for older adults who have high risks of falling and wish to improve balance and mobility.

We ask:

1. Is a 24-weeks blended supervised and self-directed technology-based DT training programme (using a mobile app) acceptable to older people at risk of falling?

2. Is a 24-weeks blended supervised and self-directed technology-based DT training programme feasible and deliverable, with potential to be adopted by falls prevention services in the NHS?

## Funding and support in kind

| **FUNDER(S)**  (Names and contact details of ALL organisations providing funding and/or support in kind for this study) | **FINANCIAL AND NON FINANCIALSUPPORT GIVEN** |
| --- | --- |
| The NIHR Research for Patient Benefit Scheme | £263,831.00 |

## Role of study sponsor and funder

The sponsor will host the study and hold negligent harm and non-negligent harm insurance policies

## Roles and responsibilities of study management committees/groups and individuals

Patient & Public Involvement Group: Ms Angela Coopper is a public co-investigator who will lead the patient and public involvement group and oversee the study to ensure outcomes meet patient benefits and interests. Participants in this study will be invited to a focus group to discuss their perspectives to the study. Discussion will be used for development of a follow-on study.

## Protocol contributors

Dr Shin-Yi Chiou, the Chief Investigator, will be responsible for the study management in all aspects. The CI will have full control of the finance.

Co-investigators will be involved in study design, patient identification and recruitment, data interpretation, and dissemination of results. A list of co-investigators: Dr Magdalena Chechlacz, Professor Afroditi Stathi, Dr Victoria Goodyear, Dr Laura Magill, Mrs Natalie Rowland (nee Ives), Ms Angela Cooper, Dr Daisy Wilson, Dr Caroline Miller, Helen Thomas, Emily Clements, and Dr Philip Kinghorn.

University of Birmingham, the sponsor, will host the study and hold negligent harm and non-negligent harm insurance policies.

Ms Angela Cooper was involved in the protocol design and will lead the PPI group and oversee the study outcomes. Participants in this study will contribute to refinement of design of a follow-on study.

KEY WORDS: Falls, mind-body training, technology, mobile applications, digital health, self-directed exercise, balance, mobility.

## Study flow chart


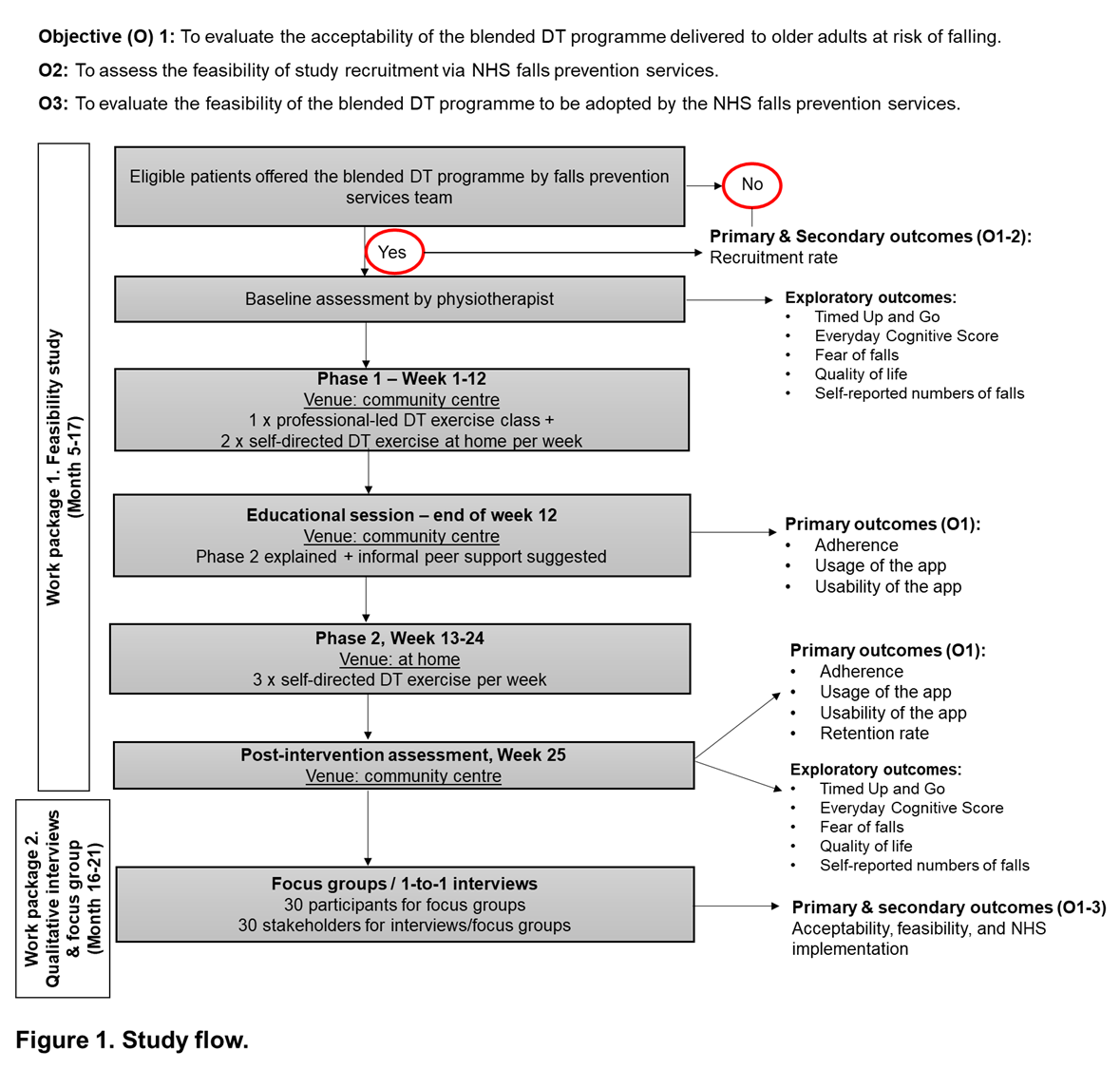


# Study protocol

1. Background

The decreasing ability to multitask is associated with increasing risk of falling in older people^1,2^. Falls are a major public health burden, with the prevalence increasing with age. Around 1 in 3 adults aged over 65, and half of people aged over 80 have at least one fall a year, and in half of such cases the falls are recurrent^3^. The most common mechanisms of major trauma or poly trauma presenting to A&E in the UK are falls^4^. Falls can lead to severe consequences such as hip fracture and loss of independence; 1/3 of individuals with a hip fracture die within a year^5^. An estimated £4.4 billion per year is spent on treating fractures and fall-related injuries^3^. Given the ageing population in the UK and the projected increase in the number of people aged 85 years and above from 1.6 million in 2016 to 3.2 million by 2036, falls prevention in older people is vital^6^.

1. Rationale

We often multitask in daily living; for example, walking while texting or reading the news while making our morning coffee. The ability to multitask, i.e., for our brain and body to work at the same time, declines with ageing. Reduced ability to multitask increases the risk of falling in older people. Research shows that training both the brain and body at the same time improves multitasking performance and mobility, thereby reducing the risk of falling^7-9^. There is evidence that some mind-body training programmes, delivered via mobile applications on tablets and smartphones, can improve mobility and the ability to multitask, and are therefore value for money^10-13^. Suitable cognitive (mind) and physical exercises can be pre-selected into a mind-body training programme, allowing people to self-direct their exercise independently while at home.

According to the conversations we had with older people who have had a fall previously and are worried about falling again, a technological, home-based exercise programme can appear daunting to older people. They have acknowledged that they may have lower levels of experience, skills, knowledge or competence in using technology, and as a result they may be reluctant to engage with such initiatives. This study proposes a blended method combining both a supervised and a self-directed mind-body training programme, with the use of technology via a mobile app. This project will examine how acceptable this new programme is likely to be to older people at risk of falling and will evaluate the feasibility of delivering it within the NHS.

1. Theoretical framework

A systematic review and meta-analysis^8^ including 30 RCTs concluded that supervised DT training in hospital or community settings (without technology) is superior to single-task (balance or resistance) training or no training for improving Timed Up and Go speed (standard mean difference [SMD] = −0.22 [95% CI:−0.38 −0.06], P = 0.007) and reducing fall rates (SMD (95% CI) = −3.03 (-4.33, -1.73), P = 0.007). Training the cognition and physical function simultaneously rather than sequentially is more beneficial^14,15^. Simultaneous cognitive and physical training can easily be delivered via technology (e.g., mobile apps), enabling professionals to select cognitive exercises that are suitable to be combined with balance exercises, allowing for the DT training to be delivered unsupervised at home. Mobile apps are interactive, provide instant feedback, and can send reminders to users. These features promote engagement and adherence to exercise^16^.

Our observational study showed improved balance after a home-based DT training programme with a mobile app in older adults without a history of falls and the adherence rate over the 6-weeks training period was 90%^12^. A recent, underpowered RCT with older people in Australia reported a trend for improvement in walking speed after a homebased DT programme via tablets^11^. These findings support the notion that technology-based exercise (not limited to DT exercise) may be a sustainable means of promoting physical activity and preventing falls in older people^17-19^. However, the evidence is not definitive in how sustained the engagement and the effectiveness of the DT training programme with technology can be. This study contributes to the need to identify a delivery method for home-based exercise using mobile apps that is acceptable to older people and has the potential to be adopted by the NHS.

1. Research question/aims
   1. Research aim

- This study aims to examine how feasible it is to deliver a blended supervised and self-directed technology-based DT training programme for older adults who have high risks of falling and wish to improve balance and mobility.
  1. Research questions
- Is a 24-weeks blended supervised and self-directed technology-based DT training programme (using a mobile app) acceptable to older people at risk of falling? Secondary research questions
- Is a 24-weeks blended supervised and self-directed technology-based DT training programme feasible and deliverable, with potential to be adopted by falls prevention services in the NHS?
  1. Outcomes

The primary outcome will be the feasibility of a subsequent phase III trial according to pre-specified progression criteria. We are primarily interested in whether the study is appealing to participants (assessed by the recruitment rate) and if the intervention is acceptable (measured by adherence and usage of the app).

The decision of whether to continue to the phase III trial will be decided by pre-defined stop-go criteria, based on the following definitions and criteria:

- Recruitment via NHS fall prevention service and in community: Defined as the number recruited/number approached (x100%). Via the planned focus groups, we will investigate whether the recruitment route chosen in this study is appropriate for the main trial.
- Adherence: Defined as the number of actual exercise days/number of exercise days offered (x100%) (60 days in total offered: 2 days/week x 12 weeks in phase 1 + 3 days/week x 12 weeks in phase 2) based on the exercise calendar.
- Usage of the app: Defined as the number of days used/number of days offered (x100%) and a self-reported online EXIT survey designed based on the feasibility assessment framework.
- Attrition: Defined as withdrawal from the study and/or no follow-up data available.

The secondary outcome measures will be: 1) Timed up and Go with and without a cognitive task, 2) everyday Cognition scales short version, 3) Falls Efficacy Scale-International, 4) Health questionnaire EQ-5D-5L, 5) Use of healthcare service via ModRUN, and 6) Self-reported numbers of falls.

1. Study design and methods of data collection and data analysis
   1. Study design

A single-arm, non-randomised feasibility study.

- 1. Recruitment

Recruitment will be carried out through the NHS falls prevention services, GP practices, and in community.

*NHS recruitment*

We will recruit from outpatient falls clinics at University Hospitals Birmingham and falls prevention services at the Birmingham Community Healthcare NHS Foundation Trust and Birmingham and Solihull Clinical Commissioning Group. These services receive patients living in different areas of Birmingham and Solihull, allowing recruitment of a diverse population. Additionally, e-searches on database of the NHS primary care in the West Midlands will be carried out by the primary care team of the CRN West Midlands. Text messages will be sent out to eligible participants.

For the site of Birmingham Community Healthcare NHS Foundation Trust and the GP practices in the West Midlands, a mail-out approach or SMS text messages will be used and carried out by an appropriate team in the Trust. Eligible participants will be identified from the database and sent a study flyer with the contact information of the research team for which they can reach out and enquire the study. Consent to enter the study will be sought at a community centre where the baseline assessment is being conducted.

For the other sites, adults meeting the eligibility criteria will be approached by a clinician in the falls clinic and provided with a study information leaflet which contains study information and contact details of the research team. The clinician may inform the research team that the participant has agreed to take part in the study and has agreed to be approached. The research team will then approach the participant to answer questions they might have and complete informed consent procedures. Alternatively, the participant can ring the research team to express willingness to take part and/or ask questions.

Furthermore, we will recruit 30 healthcare professionals working in the NHS falls prevention services in England and Wales for focus groups. We will advertise the study participation opportunity via professional bodies and social media.

Community recruitment

We will put up the study flyer in retirement homes, book clubs, etc in the West Midlands. Potential participants will be able to self-refer to members of the research team.

- 1. Planned interventions

A summary of the blended DT intervention is in Table 1:

Table 1. Summary of the blended DT exercise training programme.

|  | Supervised group exercise | Self-directed, home-based exercise |
| --- | --- | --- |
| Phase 1 – week 1-12 | **Duration:** 45 minutes  **Location:** local leisure/wellbeing centres  **Frequency:** one class/week  **Content:** 10 minutes warm-up, 30 minutes of DT training, 5 minutes cool down, followed by refreshment.  **DT training:**   - Cognitive exercises delivered via the PEAK app. - Strength and balance exercises delivered by a physiotherapist | **Duration:** 45 minutes  **Location:** at home  **Frequency:** twice/week  **Content:** 10 minutes warm-up, 30 minutes of DT training, 5 minutes cool down.  **DT training:**   - Cognitive exercises delivered via the PEAK app. - Strength and balance exercises in an exercise booklet |
| Phase 2 – week 13-24 | No supervised group exercise | **Duration:** 45 minutes  **Location:** at home  **Frequency:** three/week  **Content:** 10 minutes warm-up, 30 minutes of DT training, 5 minutes cool down.  **DT training:**   - Cognitive exercises delivered via the PEAK app. - Strength and balance exercises in an exercise booklet |

**Phase 1 – supervised group exercise 1 day/week + self-directed exercise at home 2 days/week for 12 weeks.**

Supervised group exercise.

Group exercise classes will be led by a qualified physiotherapist and delivered in the community (e.g., studios in fitness/leisure/wellbeing centres in Birmingham and Solihull).

The DT training programme requires participants to undertake concurrent cognitive and physical (balance and strength) exercise. Cognitive exercise will be delivered via a commercialised brain training app, PEAK. The content of the strength and balance exercises will align with the standard care. A study-specific account will be created for each participant so that no personal information will be shared with a third party, i.e., the app provider.

Prior to the face-to-face exercise class in week 1, all participants will receive online questionnaires to complete as their baseline data (see Outcomes below for details).

Week 1 exercise class will consist of a physical assessment of Timed Up and Go (TUG) with and without a cognitive task (see Outcomes below for details), app installation, the main DT exercise training, instruction of how to undertake the same exercises at home, and refreshment. The subsequent classes (week 2 to week 12) will consist of the DT exercise training, collecting self-reported adherence and app usage, refreshment, and individual technology support if needed.

With support from the physiotherapist, the PEAK app will be downloaded from the App Store or Google Play and installed on participants’ phones/devices. To start using the app, participants will take a cognitive assessment provided by the app and receive a brain score. The app will be set up to send notifications to the participants to remind them to exercise, supporting exercise engagement and adherence to the programme. Annual subscription fees for using the app will be purchased for all participants. This is sufficient to cover the length of the blended DT training programme (24 weeks).

In the supervised exercise class, all participants will undertake 10 minutes of strength and balance exercises alone (without a concurrent use of the app), as part of the warm-up to familiarise themselves with the movement of the exercises. This will be followed by 30 minutes of DT exercises where they will be doing the same strength and balance exercises while performing cognitive games with their device. The device will be placed on a height-adjustable music stand placed in front of the participants in an appropriate distance that allows them to use the app. The physiotherapist will provide individual support to the participants during the class to ensure everyone is able to engage with the DT exercise training. The class will be concluded with 5 minutes of cool down exercise.

At the end of the first group exercise class, the physiotherapist will provide each participant with a printout of the balance and strength exercises with pictures and instructions and instruct the participants to perform the same DT exercise training at home by themselves; a music stand will be provided to each participant. All participants will be provided an exercise calendar and shown how to document their exercise adherence outside of the exercise class. Refreshment will be provided at the end of each exercise class to promote attendance and contribute to participants’ social well-being^20,21^.

Participants will attend group exercise classes once a week for 12 weeks; their travel expenses for attending the classes will be paid. The class size will be 5-10 people.

Self-directed, home-based exercise.

Participants will be instructed to perform the same DT exercises at home on another two days per week. They can contact the research team for technical support. To record adherence, participants will be asked to tick the days that they have completed the DT exercise on their exercise calendar. The PEAK app will store data of when and which cognitive exercises are undertaken and completed. The exercise calendar will be returned to the physiotherapist or the research team at end of each month.

End of phase 1 – educational session.

Prior to the educational session, all participants will be sent an online EXIT survey (see below Outcomes section for details) and asked to complete the survey prior to the educational session.

All participants will attend an educational session for falls awareness scheduled in week 13 at the same time and same place as the exercise classes. Their travel expenses for attending the educational session will be paid. The app usage recorded from the two self-directed sessions in week 12 and the month 3 exercise calendar will be collected at the educational session. The content of the second phase of the intervention will also be explained in this session (see below for full description). Participants will be given an exercise booklet containing instructions of strength and balance exercises week-by-week and a 3-month exercise calendar. They will continue to have access to the PEAK app which will record their app usage and adherence from week 13 to 24.

Participants will be asked to consider ways that may help them stay motivated in doing the exercise in the next 12 weeks, such as creating WhatsApp groups or using Facebook Messenger, the most common messaging apps used by adults in the UK^22^, and supplemented by face-to-face “coffee shop” get-togethers to stay in touch with other participants in the same age and/or living in the same area. We will evaluate the impact of this informal peer support on engagement and sustainability of exercise as well as assess how peer support occurs in this age group, e.g., mostly in person or via social media in the planned focus groups (see below Qualitative interviews and focus groups section for details).

**Phase 2 – self-directed, home-based DT training programme 3 days/week for 12 weeks.**

Participants will undertake 30 minutes of self-directed DT exercises at home 3 times per week for 12 weeks. They will perform the DT exercise using the PEAK app and the strength and balance exercises in the exercise booklet. Usage and engagement of the app of the participants will continue to be recorded by the app. Participants will record their exercise adherence in the exercise calendar. Participants can contact the research team for technical assistance.

All participants will be contacted in week 24 to attend a post-assessment session in week 25. Their travel expenses for attending the post-assessment will be met.

End of Phase 2 (Week 25) – post assessment and app data extraction session.

Participants will be invited back to the same place of the exercise classes to undergo the TUG assessment. The research team will perform the TUG assessment and document the app usage data from week 13 to week 24 from participants’ phones/devices. The exercise calendars will also be collected from the participants. All participants will be sent the same online questionnaires as they completed at baseline and at the end of the phase 1 and asked to complete the questionnaires within 2 weeks from the post-assessment.

- **Qualitative interviews and focus groups**

Five face-to-face focus groups (FGs) with a total of 30 participants who have completed the programme (4-6 participants per group for 45-60 minutes) will be conducted. We will purposefully sample 30 participants, based on the demographic information, geographic location, exercise adherence, and results of the EXIT survey, to attend the focus groups.

Additionally, we will conduct five online FGs with a total of 30 healthcare professionals, including GPs, district nurses, paramedics, and physiotherapists who are part of the NHS falls prevention care pathways in Birmingham and Solihull, via MS Teams. Clinicians and physiotherapists involved in this study will be invited to take part.

The purpose of conducting the FGs is to allow for in-depth exploration of the results obtained from the feasibility study as well as to capture attributes of the acceptability and feasibility which are unable to be recorded by outcome measures stated in the feasibility study above. Questions will be developed based on the framework for qualitative research in feasibility studies for trials^23^ and input from the PPI group. Specifically, questions will cover 4 main categories:

- - Intervention content and delivery
  - Study design, conduct and processes
  - Outcomes (e.g., are they important to service users?)
  - Measures (e.g., are the process valid for the service users?)

Descriptive data from the adherence, usage of the app, and the EXIT survey will be used as prompts. The FG will be led by two members from the study team. The reason to have two people leading the FGs is to ensure both verbal and non-verbal interactions and group dynamics during the discussions can be documented^40^.

FGs will be voice recorded and later transcribed and analysed using a deductive thematic approach with Nvivo9 software; data for the study participants and the stakeholders will be analysed separately.

- 1. Assessments

**Primary outcomes:** Whether the study is appealing to participants (assessed by the recruitment rate) and if the intervention is acceptable (measured by adherence and usage of the app).

1. Recruitment via NHS falls prevention services and in community. The recruitment rate [(number recruited/number approached) x100%] will be assessed. Via the planned focus groups, we will investigate whether the recruitment route chosen in this study is appropriate for the main trial.

1. Usage of the app during the 24-weeks blended DT training programme:
   1. Numbers of days undertaking both cognitive and physical exercises and numbers of exercises completed, assessed via data extracted from the PEAK app cloud and recorded on the exercise calendars. App-recorded and self-reported adherence to the DT exercise will be compared to inform how to best record exercise adherence in the full trial.
   2. Types of cognitive exercises completed with the physical exercises, evaluated via the data extracted from the PEAK app, to provide information on content design of the DT training programme in the future.
2. Study retention rate: [(number completed study/ number recruited) x100%] will be assessed.
3. Usability, perceived effectiveness and satisfaction from a self-reported online EXIT survey based on the feasibility assessment framework (Technical, Economic, Legal, Operational, and Schedule)^24^. Data will be collected at the end of the phase 1 and 2. Exampled questions are as follows:
   - Overall, how beneficial did you find the training to be?
   - How easy was it to fit the training in to your daily routine?
   - Which of the following did you enjoy about the app?
   - Which of the following limited your use of the app?
   - How happy were you with the level of improvement of balance between the start and finish?
   - Have you noticed any other physical benefits or improvement after completing 6 months of the blended DT training?
   - Were you happy with the way your data being stored and collected by the PEAK?
   - Do you think the blended DT training programme with PEAK is a good way of achieving the above?
   - Did you receive the support you needed during the programme?
   - How likely will you continue with the training after the study completion?
   - If you were to continue with the training, how regularly do you think you would do it?
   - What could we have changed or improved?

The EXIT survey will be an online survey which will be uploaded through REDCap, which is secure encrypted data management software. We will use the REDCap managed by the Birmingham Centre for Observational and Prospective Studies (BiCOPS).

**Secondary outcome measures**

The following outcome data will be collected at two time points: before and after the 24-weeks programme. Data will be collected within 2 weeks post-programme.

1. Timed Up and Go with and without a cognitive task (TUG) will be assessed in-person in the same place where the exercise classes will be held.

For the TUG alone, participants will be asked to rise from a standard armchair, walk to a marker 3 meter away, turn, walk back, and sit down again. For the TUG with cognitive tasks (TUG Cognitive), participants will be asked to count backwards in sevens from a random start point while completing the TUG. Each condition will repeat 3 times. The best performance will be included in datasheet.

Additionally, the participants will be invited to take part in a virtual TUG assessment via Zoom after their in-person post assessment. The virtual assessment will be scheduled to be close to the in-person assessment to provide accurate validation measures. Participants will be informed that this is for validation purposes and is optional.

The following self-reported questionnaires will be collected online via REDCap.

1. Everyday Cognition scales short version (ECog-12; 12 questions in total) is an informant-rated questionnaire designed to detect cognitive and functional decline linking to independence in the activities of daily living^36^. Results will inform any effect of the training programme on cognitive function affecting daily living which declines with ageing.
2. Falls Efficacy Scale-International is a valid and reliable questionnaire (6 items, max score: 64) for assessing confidence in the performance of activities of daily living and in levels of fall risk (>24 points)^37^.
3. EQ-5D-5L questionnaire will be used to assess the quality of life^38^ and to test its suitability to be used in the main trial.
4. ModRum for the use of healthcare services for collecting health economics information.
5. Self-reported numbers of falls during the study period will be recorded.

Our data collection tools and plan were presented to the PPI events where members agreed the numbers and the frequency of the data collection were acceptable and not burdensome

- 1. Statistical analysis

The analysis undertaken within this feasibility study will mainly be descriptive. The progression criteria in the stop-go will be summarised as proportions and percentages with 95% confidence intervals. The numbers of days of exercise and the numbers of exercises completed recorded in the app and on the exercise calendars during the 6-month intervention period will be summarised to describe the usage of the app and adherence. To understand any change in the usage of the app between phase 1 and phase 2, we will assess the app usage in phase 1 and phase 2 separately. Information from the EXIT survey will be summarised.

Outcome measure data will be summarised at baseline and follow-up using appropriate summary statistics. Exploratory analysis may compare the data from the two time points using a paired t-test (depending on the distribution of the data) to provide preliminary data on the effects of the blended intervention.

1. Study setting

Participant identification will be carried out in falls clinics at University Hospitals Birmingham (Queen Elizabeth Hospital Birmingham, Birmingham Heartlands Hospital, and Solihull Hospital) and falls prevention services at the Birmingham Community Healthcare NHS Foundation Trust and Birmingham and Solihull Clinical Commissioning Group.

Participant recruitment will be conducted in two ways: 1) a mail-out approach for the Birmingham Community Healthcare NHS Foundation Trust, and 2) by clinicians from the University Hospitals Birmingham and Birmingham and Solihull Clinical Commissioning Group.

Study consent will be performed either at the clinics or in the community by the study team. Members of the team who will be carrying out study consent and data collection will undergo GCP training.

Interventions and assessments will be conducted in the community by the research team.

1. Participant recruitment

Adults aged 65 years and above who have fallen more than once in the past 12 months and are referred to undertake exercise for managing their fall risks.

- 1. Eligibility Criteria
     1. Inclusion criteria

Patients will be recruited if they:

1. Aged 65 years and above,
2. Can give informed consent,
3. Demonstrate sufficient cognition/hearing/vision to follow instructions of the assessment and the exercise programme,
4. Can stand with one hand support on the current walking aid for at least 60s,
5. Can stand up from a chair independently and walk for 6 meters independently with the current walking aid,
6. Are self-toileting,
7. Own or have access to a smartphone/iPad/iPod touch/tablet, and
8. Have fallen more than once in the last 12 months.
   - 1. Exclusion criteria

Patients will be excluded if they:

1. have an unstable or acute medical condition that precluded exercise participation,
2. suffer from a progressive neurological condition (such as Parkinson’s disease or multiple sclerosis),
3. are not recommended to undertake any forms of exercise by their GPs or secondary healthcare team; for example, having uncontrolled blood pressure, postural hypotension, acute/unstable cardiac issues, and dizziness brought on by exercise or changing posture. or
4. are currently participating in a different research study for managing their fall risks.

Additionally, the inclusion criteria for recruiting stakeholders to the qualitative focus group study are:

- - Aged 18 years and above,
  - A healthcare professional in the NHS falls prevention care pathways in the Birmingham and Solihull.

Healthcare professionals with less than 6 months experience in the falls prevention care pathways will not be recruited.

- 1. Recruitment target
     1. Size of recruitment target

A sample size of 50 was chosen based on published literature where a sample size of between 50 and 100 participants (total; i.e. 25 to 50 per group) is recommended for pilot studies42-44. If we identify 100 eligible participants, we will be able to estimate a participation rate of 50% (green stop-go; see below) within a 95% confidence interval of +/-9.8%. With a sample size of 50 participants, we will be able to estimate an attrition rate of 20% (green stop-go; see below) to within a 95% confidence interval of +/-11%.

- - 1. Recruitment technique

Clinicians in the falls team will screen and identify eligible participants for the study. The potential participants will be provided a study flyer with contact information of the research team to enquire study information if they wish. Clinicians or research nurses in the NHS sites may mail out study flyers or send out text messages to eligible participants who will be able to self-identified themselves and contact the research team.

- 1. Recruitment
     1. Participant identification

Identification of suitable participants will be carried out through falls clinics at the University Hospitals Birmingham and falls prevention services at the Birmingham Community Healthcare NHS Foundation Trust and Birmingham and Solihull Clinical Commissioning Group.

- - 1. Consent

Consent to enter the study will be sought from each participant by members of the research team who have completed a GCP training at the clinic or in the community after a full explanation has been given, an information leaflet offered, and time allowed for consideration. Signed participant consent will be obtained. The right of the participant to withdraw from the study without giving reasons will be respected. All participants are free to withdraw at any time from the protocol treatment without giving reasons and without prejudicing further treatment; this will be made clear to all the participants given that they might be suggested by their clinicians to take part in the study.

All participants will be allocated a unique ID at the point of consent and all provided data will be stored with that ID. A separate file (paper-based or electronic) will link the participants to the ID. The file will be encrypted and stored on closed MS Teams channel which will be managed by the CI and only members of the research team have access to.

1. Safety reporting

Definitions

Adverse Event (AE): any untoward medical occurrence in a patient or clinical study subject.

Serious Adverse Event (SAE): any untoward and unexpected medical occurrence or effect that:

• Results in death

• Is life-threatening – refers to an event in which the subject was at risk of death at the time of the event; it does not refer to an event which hypothetically might have caused death if it were more severe

• Requires hospitalisation, or prolongation of existing inpatients’ hospitalisation

• Results in persistent or significant disability or incapacity

• Is a congenital anomaly or birth defect

Medical judgement should be exercised in deciding whether an AE is serious in other situations. Important AEs that are not immediately life-threatening or do not result in death or hospitalisation but may jeopardise the subject or may require intervention to prevent one of the other outcomes listed in the definition above, should also be considered serious.

Reporting Procedures

All adverse events should be documented and assessed; those events identified as reportable must be reported in accordance with the sponsor’s safety reporting procedures. Depending on the nature of the event the reporting procedures below should be followed. Any questions concerning adverse event reporting should be directed to the Chief Investigator in the first instance.

Non-serious AEs: All such events, whether expected or not, should be recorded

Serious AEs: An SAE form should be completed and email to the Chief Investigator within 24 hours. However, relapse and death due to a pre-existing condition, and hospitalisations for elective treatment of a pre-existing condition do not need reporting as SAEs. All SAEs assessed as reportable should be reported to the REC within 15 days where in the opinion of the Chief Investigator, the event was:

• ‘related’, ie resulted from the administration of any of the research procedures; and

• ‘unexpected’, ie an event that is not listed in the protocol as an expected occurrence

Reports of related and unexpected SAEs should be submitted within 15 days of the Chief Investigator becoming aware of the event, using the NRES SAE form for non-IMP studies. The Chief Investigator must also notify the Sponsor of all reportable SAEs in accordance with the sponsor’s safety reporting procedures under the Quality Management System (QMS; https://www.birmingham.ac.uk/research/activity/mds/mds-rkto/governance/qms.aspx).

Local investigators should report any SAEs as required by their Local Research Ethics Committee, Sponsor and/or Research & Development Office.

Contact details for reporting SAEs

Please send SAE forms to:

**Dr Shin-Yi Chiou**

**School of Sport Exercise and Rehabilitation Sciences,**

**University of Birmingham, Edgbaston,**

**Birmingham, UK. B15 2TT.**

1. Data Handling and Record Keeping

Source data

Source data is defined as all information in original records and certified copies of original records of clinical findings, observations, or other activities in a clinical study necessary for the reconstruction and evaluation of the study.

Source data within this study will be kept as part of the participants’ study notes generated and maintained by the research team.

Most of the follow-up within this study is via patient-reported questionnaires; these are via validated questionnaires and through additional patient reported outcomes (e.g., numbers of falls). This data is collected electronically and inputted directly by the participants onto the bespoke study specific REDCap database system. The data inputted onto the study REDCap database forms the source data.

Data Management

Processes will be employed to facilitate the accuracy and completeness of the data included in the final study report. These processes will be detailed in the study specific Data Management Plan.

Data entry will be completed via a bespoke BiCOPS REDCaP study database. The data capture system will conduct automatic range checks for specific data values to ensure high levels of data quality. Queries and requests for missing clinical data will be raised using data clarification forms (DCFs) via the study database, with the expectation that these queries will be completed by the research team within 30 days of receipt.

Participant completed questionnaires at each of the follow-up timepoints cannot be queried. Participants will be contacted up to three times to request completion of missing questionnaires.

### Data collection

Data collection within the Study is limited.

All clinical data collected by participating sites will be collected by the research team at site and entered directly onto the REDCap database.

It is the responsibility of the local PI to ensure the accuracy of all data entered. The Study Delegation Log will identify all those personnel with responsibilities for data collection.

### Participant completed questionnaires

Participant completed questionnaires will be completed online, although they may be completed by post or telephone according to participant preference.

At the time of consent and entry into the study, what the study involves will be discussed with each participant. It will be explained that completion of questionnaires plus other patient-reported outcomes will be requested at three timepoints, at baseline, end of phase 1, and at 24-weeks post-completion of the intervention and the methods of completion of the questionnaires discussed.

Missing patient-reported data cannot be chased.

Participants who have not returned completed forms will be contacted up to 3 times after the due date to request completion of the questionnaires. After this time, unreturned forms will be regarded as missing and will not be requested again.

### Data security

UoB has policies in place, which are designed to protect the security, accuracy, integrity and confidentiality of Personal Data. The study will be registered with the Data Protection Officer at UoB and will hold data in accordance with the Data Protection Act (2018 and subsequent amendments). The Study Office has arrangements in place for the secure storage and processing of the study data which comply with UoB policies.

The Study Database System incorporates the following security countermeasures:

**Physical security measures:** restricted access to the building, supervised onsite repairs and storages of back-up tapes/disks are stored in a fire-proof safe.

**Logical measures for access control and privilege management:** including restricted accessibility, access controlled servers, separate controls of non-identifiable data.

**Network security measures:** including site firewalls, antivirus software and separate secure network protected hosting.

**System management:** the system will be developed by the BiCOPS Team at the CHAPTER Study Office, and will be implemented and maintained by the BiCOPS Team.

**System design:** the system will comprise of a database and a data entry application with firewalls, restricted access, encryption and role-based security controls.

**Operational processes:** the data will be processed and stored within BiCOPS.

**Data Protection Registration:** UoB’s Data Protection Registration number is Z6195856.

### Archiving

It is the responsibility of the PI to ensure all essential study documentation and source documents (e.g., signed ICFs, Investigator Site Files, participants’ hospital notes, copies of CRFs) at their site are securely retained for the contractual period. Archiving will be authorised by BiCOPS on behalf of the sponsor following submission of the end of study report. No documents should be destroyed without prior approval from the BiCOPS Director.

The electronic TMF will be stored at BiCOPS for at least 3 years after the end of the study. Long-term offsite data archiving facilities will be considered for storage after this time; data will be stored securely and confidentially for at least 10 years.

1. Ethical and regulatory considerations
   1. Assessment and management of risk

1.Patients may feel that their participation or non-participation in this study will affect the standard of care they receive. The patients will be informed verbally and in the Patient Information Sheet that their participation in the study is entirely voluntary and will not affect their care or treatment in any way.

2. Patients may feel they need time to consider their participation and would like to discuss with other people. The patients will be given the information sheet with our contact details and will have adequate time to decide and contact us if they would like to participate in the study.

3. Due to the nature of the exercise being to challenge balance, there is a risk that participants may lose balance during the exercise. There will be always a physiotherapist and an assistant present during the classes to ensure safety of the participants. Additionally, instructions on how to exercise safely will be explained to the participants; for example, exercise near a wall or a kitchen top will reduce risks of falling.

4. Conditions that were previously unknown to the participant may be identified during the process of research data collection (e.g., pain during a certain movement); this could be considered both a beneficial and unfavourable consequence. In the case of potentially significant findings, these will be explained to the participant, including the potential consequences and further investigations or treatment that might be indicated. These findings will be relayed to the participants’ General Practitioners (GP) or secondary care medical team (e.g., Fall Prevention Team), who will be advised to arrange further management or to refer to appropriate specialties; this is usual practice in the United Kingdom (UK). Specific consent for findings to be communicated with their GP or medical team will be gained from the participant; the GP will be informed routinely of recruitment to this trial. It should be clarified to the participant that any investigations or management are separate from the research trial and are part of NHS care.

5. Other key ethical issues revolve around data protection and confidentiality and appropriate measures will be taken to ensure this is maintained for all volunteers. No identifiable data will be recorded on datasheets during the participation. Only named investigators will have access to the study data. All electronic data will be anonymised and kept separately from their personal information.

6. Participants may experience challenges in managing complex technology. They will have face-to-face support from the physio and research team in Phase 1 to help them overcome potential barriers to use of technology. They can contact the research team for technical assistant throughout the study. Note that evaluating the reaction of older people to the use of technology for health reasons is an objective of this feasibility study.

7. Participants may find the brain training tasks difficult and feel demotivated. We will help the participants choose appropriate cognitive tasks paired with the physical exercise that suit their ability. As they progress, the tasks can then be modified.

8. Current government guidelines and local safety procedures in relation to the ongoing pandemic will be adhered to in order to mitigate the risk of exposure for both the participants and researchers involved in the study.

9. There are risks to researchers themselves when conducting interventions and assessments outside the University, e.g., in a community leisure centre, University of Birmingham Health and Safety guidance on Field Work will be followed:
<https://intranet.birmingham.ac.uk/hr/documents/public/hsu/hsuguidance/9HSGG.pdf>

- 1. Research Ethics Committee (REC) and other Regulatory review & reports

The study will be reviewed by the NRES Committee and Health Research Authority (HRA).

Before the start of the study, a favourable opinion will be sought from a REC for the study protocol, informed consent forms and other relevant documents e.g. advertisements.

The Chief Investigator will produce the annual reports as required and notify the REC of the end of the study. An annual progress report (APR) will be submitted to the REC and study sponsor within 30 days of the anniversary date on which the favourable opinion was given, and annually until the study is declared ended. If the study is ended prematurely, the Chief Investigator will notify the REC, including the reasons for the premature termination. Within one year after the end of the study, the Chief Investigator will submit a final report with the results, including any publications/abstracts, to the REC.

### Regulatory Review & Compliance

Before any site can enrol patients into the study, the Chief Investigator will ensure that appropriate approvals from participating organisations are in place.

### Amendments

The sponsor must be notified of all amendments to the protocol, both substantial and non-substantial. Review of amendments by the sponsor will act as the confirmation that the sponsor confirms approval of the amended protocol.

- 1. Peer review

The protocol was reviewed by the NIHR Research for Patient Benefit sub-committee as part of funding decision-making process.

- 1. Patient & Public Involvement

Users were involved in protocol design during the bid development. The PPI lead Ms Cooper will be involved throughout the study to ensure outcomes meet the needs of the patients and the public. Focus groups data will be used to inform design and development of future trials.

- 1. Protocol compliance

The Chief Investigator has the responsibility to ensure that the research will act in compliance with the approved protocol. Researchers involved in study consent and data collection of the study will have to undertake the Good Clinical Practice (GCP) training.

Accidental protocol deviations can happen at any time. They must be adequately documented on the relevant forms and reported to the Chief Investigator and Sponsor immediately.

Deviations from the protocol occur from time to time, those deviations which are found to frequently recur will be documented and reviewed, corrective and preventative measures will be put in place as required.

Any deviations from the protocol or GCP standards that are found to likely effect to a significant degree; the safety or physical or mental integrity of the participants or the scientific value of the study will be classed as a serious breach. In the event that a serious breach is identified the University of Birmingham’s serious breach reporting procedures will be followed and the breach will be reported to the sponsor and REC in accordance with requirements.

- 1. Data protection and patient confidentiality

The Chief Investigator has the responsibility to ensure that participant anonymity is protected and maintained. They must also ensure that their identities are protected from any unauthorised parties. Information with regards to study participants will be kept confidential and managed in accordance with the Data Protection Act 2018, General Data Protection Regulation (GDPR), The UK Policy for Health and Social Care and Research Ethics Committee Approval.

All participants will be allocated a study ID at point of consent. The Consent forms will be stored in a secure cabinet by the research team at the prospective sites. Anonymized data will be stored on University computers.

Personal data required to contact patients will be stored on the secure University server (i.e., Bear RDS) that is password protected and meets University of Birmingham criteria for storing personal data. This information will normally be kept for 10 years as per University of Birmingham policy; however, contact details such as telephone numbers and email addresses will be deleted after the study is completed and will not be stored for 10 years.

Data generated by this study will be kept for 10 years as per University of Birmingham guidelines including the patient consent forms. This data will be appropriately destroyed after 10 years.

Only members of the research team will have access to the information and data collected. The Chief investigator will be the custodian of the data, which will be stored in a password-protected computer at University of Birmingham.

The subjects will be anonymised with regards to any future publications relating to this study.

- 1. Indemnity

The University of Birmingham has in place Clinical Trials indemnity coverage for this trial which provides cover to the University for harm which comes about through the University’s, or its staff’s, negligence in relation to the design or management of the trial and may alternatively, and at the University’s discretion provide cover for non-negligent harm to participants.

With respect to the conduct of the trial at Site and other clinical care of the patient, responsibility remains with the NHS organisation responsible for the clinical site and is therefore indemnified through NHS Resolution. The NHS have a duty of care to participants whether or not the participant is taking part in a clinical study.

- 1. End of study and archiving

The study will be ended when 1) 50 participants complete the intervention and assessments. All data will be retained for a minimum of 10 years as per University of Birmingham policy.

- 1. Access to the final study dataset

Only members of the research team will have access to the information and data collected. The Chief investigator will be the custodian of the data, which will be stored in a password-protected computer at University of Birmingham. Identity the individuals involved in the study who will have access to the full dataset.

- 1. Dissemination policy

All presentations and publications pertaining to this study require authorisation from the Chief Investigator, who is responsible for the intellectual property arising from this study.

The Chief Investigator will review submissions for publication. Any publications or presentations relating to this study will be submitted in accordance with University of Birmingham policy.

Results of this study will be available to participants on request.

- 1. Authorship eligibility guidelines and any intended use of professional writers

The Chief Investigator will be the corresponding author on the final study report and publications. Co-investigators and researchers who are actively involved in data interpretations and manuscript writing will be listed as authors on the study report and publications.

1. References
2. Muir-Hunter, S. W. & Wittwer, J. E. Dual-task testing to predict falls in community-dwelling older adults: a systematic review. Physiotherapy 102, 29-40 (2016). https://doi.org:10.1016/j.physio.2015.04.011
3. Bayot, M. et al. Can dual-task paradigms predict Falls better than single task? - A systematic literature review. Neurophysiol Clin 50, 401-440 (2020). https://doi.org:10.1016/j.neucli.2020.10.008
4. The Public Health England. Falls: applying All Our Health, <https://www.gov.uk/government/publications/falls-applying-all-our-health/falls-applying-all-our-health> (2022).
5. Kehoe, A., Smith, J. E., Edwards, A., Yates, D. & Lecky, F. The changing face of major trauma in the UK. Emerg Med J 32, 911-915 (2015). https://doi.org:10.1136/emermed-2015-205265
6. Downey C, Kelly M, Quinlan JF. Changing trends in the mortality rate at 1-year post hip fracture - a systematic review. World J Orthop. 2019 Mar 18;10(3):166-175. doi: 10.5312/wjo.v10.i3.166.
7. Chief Medical Officer’s Annual Report 2023 Health in an Ageing Society. https://assets.publishing.service.gov.uk/media/65562ff2d03a8d000d07faa6/chief-medical-officers-annual-report-2023-web-accessible.pdf
8. Schoene, D., Valenzuela, T., Lord, S. R. & de Bruin, E. D. The effect of interactive cognitive-motor training in reducing fall risk in older people: a systematic review. BMC Geriatr 14, 107 (2014). https://doi.org:10.1186/1471-2318-14-107
9. Wang, X. et al. Cognitive motor interference for preventing falls in older adults: a systematic review and meta-analysis of randomised controlled trials. Age Ageing 44, 205-212 (2015). https://doi.org:10.1093/ageing/afu175
10. Varela-Vasquez, L. A., Minobes-Molina, E. & Jerez-Roig, J. Dual-task exercises in older adults: A structured review of current literature. J Frailty Sarcopenia Falls 5, 31-37 (2020). https://doi.org:10.22540/JFSF-05-031
11. Wang, R. Y., Huang, Y. C., Zhou, J. H., Cheng, S. J. & Yang, Y. R. Effects of Exergame-Based Dual-Task Training on Executive Function and Dual-Task Performance in Community-Dwelling Older People: A Randomized-Controlled Trial. Games Health J 10, 347-354 (2021). https://doi.org:10.1089/g4h.2021.0057
12. Callisaya, M. L. et al. A novel cognitive-motor exercise program delivered via a tablet to improve mobility in older people with cognitive impairment - StandingTall Cognition and Mobility. Exp Gerontol 152, 111434 (2021). https://doi.org:10.1016/j.exger.2021.111434
13. Papi, E., Chiou, S. Y. & McGregor, A. H. Feasibility and acceptability study on the use of a smartphone application to facilitate balance training in the ageing population. BMJ Open 10, e039054 (2020). https://doi.org:10.1136/bmjopen-2020-039054
14. Tuena C, Borghesi F, Bruni F, Cavedoni S, Maestri S, Riva G, Tettamanti M, Liperoti R, Rossi L, Ferrarin M, Stramba-Badiale M. Technology-Assisted Cognitive Motor Dual-Task Rehabilitation in Chronic Age-Related Conditions: Systematic Review. J Med Internet Res. 2023 May 22;25:e44484. doi: 10.2196/44484. Erratum in: J Med Internet Res. 2023 Sep 26;25:e51591.
15. Fraser, S. A. et al. Does Combined Physical and Cognitive Training Improve Dual-Task Balance and Gait Outcomes in Sedentary Older Adults? Front Hum Neurosci 10, 688 (2016). https://doi.org:10.3389/fnhum.2016.00688
16. Sipila, S. et al. Effects of physical and cognitive training on gait speed and cognition in older adults: A randomized controlled trial. Scand J Med Sci Sports 31, 1518-1533 (2021). https://doi.org:10.1111/sms.13960
17. Sullivan, A. N. & Lachman, M. E. Behavior Change with Fitness Technology in Sedentary Adults: A Review of the Evidence for Increasing Physical Activity. Front Public Health 4, 289 (2016). https://doi.org:10.3389/fpubh.2016.00289
18. Valenzuela, T., Okubo, Y., Woodbury, A., Lord, S. R. & Delbaere, K. Adherence to Technology-Based Exercise Programs in Older Adults: A Systematic Review. J Geriatr Phys Ther 41, 49-61 (2018). https://doi.org:10.1519/JPT.0000000000000095
19. Netz, Y. et al. Personalized Exercise Programs Based upon Remote Assessment of Motor Fitness: A Pilot Study among Healthy People Aged 65 Years and Older. Gerontology, 1-15 (2021). https://doi.org:10.1159/000517918
20. Smith-Ray, R. L., Makowski-Woidan, B. & Hughes, S. L. A randomized trial to measure the impact of a community-based cognitive training intervention on balance and gait in cognitively intact Black older adults. Health Educ Behav 41, 62S-69S (2014). https://doi.org:10.1177/1090198114537068
21. Stathi, A. et al. Effect of a physical activity and behaviour maintenance programme on functional mobility decline in older adults: the REACT (Retirement in Action) randomised controlled trial. Lancet Public Health 7, e316-e326 (2022). https://doi.org:10.1016/S2468-2667(22)00004-4
22. Burton, E. et al. Encouraging Adults Aged 65 and over to Participate in Resistance Training by Linking Them with a Peer: A Pilot Study. Int J Environ Res Public Health 20 (2023). https://doi.org:10.3390/ijerph20043248
23. Ofcom. Adults' Media Use and Attitudes report 2022.
24. O'Cathain, A. et al. Maximising the impact of qualitative research in feasibility studies for randomised controlled trials: guidance for researchers. Pilot Feasibility Stud 1, 32 (2015). https://doi.org:10.1186/s40814-015-0026-y
25. Avan, B. I., Berhanu, D., Umar, N., Wickremasinghe, D. & Schellenberg, J. District decision-making for health in low-income settings: a feasibility study of a data-informed platform for health in India, Nigeria and Ethiopia. Health Policy Plan 31 Suppl 2, ii3-ii11 (2016). https://doi.org:10.1093/heapol/czw082
26. Appendices
    1. Appendix 1- Required documentation

List here all the local documentation you require prior to initiating a participating site (e.g. CVs of the research

- CI’s CV
- Patient Information Sheet
- Consent form
- Study flyer
- Questionnaires (used by the researchers)
- Invitation letter
- GP letter
- Instruction of arm cycling exercise at home
  1. Appendix 2 – Schedule of Procedures
  2. Appendix 3 – Amendment History

Amendment No. Protocol version no. Date issued Author(s) of changes Details of changes made

List details of all protocol amendments here whenever a new version of the protocol is produced.

Protocol amendments must be submitted to the Sponsor for approval prior to submission to the REC.
